# Supplementary material for: External validation and recalibration of an incidental meningioma prognostic model – IMPACT: protocol for an international multicentre retrospective cohort study
Source: BMJ Open. 2022 Jan 18;12(1):e052705. doi: 10.1136/bmjopen-2021-052705 (PMC8768908; doi:10.1136/bmjopen-2021-052705)
Supplement: Supplementary data [file bmjopen-2021-052705supp001.pdf]

1 APPENDICES

1.1. Appendix. 1. Guide for REDCap database developers

Characteristic	Options	Notes
**BASELINE CLINICAL CHARACTERISTICS (PAGE/SECTION 1)**		
Age (years)	Free field	Required entry
Sex	Dropdown list/check box<br>• Male<br>• Female	
Ethnicity	Dropdown list/check box<br>• White<br>• Mixed / Multiple ethnic groups<br>• Asian / Asian British<br>• Black / African / Caribbean / Black British<br>• Other ethnic group<br>• NA	Required entry. Allow one option only.<br>Other ethic group prompts a free text box
Comorbidities	Check box<br>• Hypertension - systolic > 140 or diastolic > 90 and patients on medical treatment<br>• Previous myocardial infarction<br>• Congestive heart failure<br>• Peripheral vascular disease	Required entry. Allow multiple options.<br>Note to data collector: Age and comorbidities will be used to calculate the age adjusted Charlson comorbidity index. Each of these is defined in the guide provided

	• Previous stroke/TIA - If hemiplegia present, do not check<br>• Hemi/paraplegia<br>• Diabetes which requires medical treatment<br>• Diabetes with end-organ damage - if so, do not check diabetes that requires treatment<br>• COPD/Asthma<br>• Renal disease<br>• Mild liver disease - Hep B/C or cirrhosis without portal hypertension<br>• Moderate to severe liver disease - cirrhosis with portal hypertension, jaundice, ascites<br>• Peptic ulcer disease<br>• Cancer - excluding basal cell carcinoma<br>• Metastatic cancer - if so, do not check cancer<br>• Rheumatic or connective tissue disease<br>• HIV/AIDS<br>• Skin ulcers/cellulitis<br>• Depression<br>• Dementia<br>• On Warfarin	
WHO Performance status	Dropdown list/check box<br><br>• 0<br>• 1<br>• 2	Required entry. Allow one option only

	• 3<br>• 4	
Indication for scan	Dropdown list/check box<br><br>• Headache<br>• Cerebrovascular accident<br>• Head injury<br>• Audiovestibular symptoms<br>• Visual symptoms<br>• Psychiatric symptoms<br>• Cognitive symptoms<br>• Loss of consciousness<br>• Other	Required entry. Allow multiple options.<br><br>Note to data collector: The meningioma must not be thought to be the cause of these symptoms. Other indications might include lethargy, research, sinusitis, anosmia…etc.<br><br>Other prompts a free text box
**NUMBER OF MENINGIOMAS ON 1ST DIAGNOSTIC SCAN (PAGE/SECTION 2)**		
Initial scan date	DD/MM/YYYY	Required entry. YYYY can't be ≤2007 or ≥2011
How many meningiomas?	Check box<br><br>• Single<br>• Multiple	Required entry<br><br>If a single meningioma, direct to section/page 3.<br><br>If multiple, prompt a new entry point (e.g. check box with options being 2-6) with number of meningiomas present. Each meningioma will then be treated as a separate entity with regards to the upcoming sections.
**BASELINE IMAGING CHARACTERISTICS (SECTION/PAGE 3)**		
Meningioma signal intensity on T2	Dropdown list/check box<br><br>• Hypointense<br>• Hyperintense<br>• Isointense<br>• NA	Required entry. Allow one option only<br><br>Note to data collector: In relation to the contralateral grey matter. If only baseline CT available, NA

Meningioma signal intensity on FLAIR	Dropdown list/check box<br><br>• Hypointense<br>• Hyperintense<br>• Isointense<br>• NA	Required entry. Allow one option only<br><br>Note to data collector: In relation to the contralateral grey matter. If only baseline CT available, NA
Peritumoural signal intensity on T2	Dropdown list/check box<br><br>• 0-5%<br>• 6-33%<br>• 34-66%<br>• 67-100%<br>• NA	Required entry. Allow one option only<br><br>Note to data collector: In relation to tumour volume. If only baseline CT available, NA
Peritumoural signal intensity on FLAIR	Dropdown list:<br><br>• 0-5%<br>• 6-33%<br>• 34-66%<br>• 67-100%<br>• NA	Required entry. Allow one option only<br><br>Note to data collector: In relation to tumour volume. If only baseline CT available, NA
Venous sinus nearby	Checkbox	Required entry.<br><br>Note to data collector: Tick box if within 10 mm, in direct contact or invading one of: superior sagittal sinus (SSS), sigmoid sinus (SS), transverse sinus (TS), cavernous sinus (CS) and the confluence of sinuses (CoS)
If yes, specify	Dropdown list/check box<br><br>• Superior sagittal sinus<br>• Cavernous sinus<br>• Sigmoid sinus<br>• Transverse sinus<br>• Confluence of sinuses	Prompt entry if previous option (venous sinus nearby) has been ticked. Allow one option only.
Separate, direct contact or invaded?	Dropdown list/check box<br><br>• Separate	Prompt entry if previous option (venous sinus nearby) has been ticked. Allow one option only

	• Direct contact<br>• Invaded	
In contact with critical neuro-vascular structures?	Checkbox	Required entry
If yes, which	Dropdown list/check box<br><br>• Internal carotid artery<br>• Basilar artery<br>• Vertebral artery<br>• Middle cerebral artery<br>• Anterior cerebral artery<br>• Posterior cerebral artery<br>• Optic apparatus (optic nerve and chiasm)<br>• Trigeminal nerve<br>• Facial nerve<br>• Vestibulo-cochlear nerve<br>• Other	Prompt entry if previous option (In contact with critical neuro-vascular structures?) has been ticked
Major axis (mm)	Free field	Required entry<br><br>Note to data collector: In mm to 1 dp. Measure on axial T1/CT + GAD
Minor axis (mm)	Free field	Required entry<br><br>Note to data collector: In mm to 1 dp. Perpendicular to the major axis on axial T1/CT + GAD
Cor/sag major axis	Free field	Require entry<br><br>Note to data collector: In mm to 1 dp. Maximum height
Location	Dropdown list/Check box<br><br>• Convexity<br>• Parasagittal<br>• Parafalcine	Required entry. Allow one option only<br><br>Note to data collector: as per ICOM classification (appendix 2)

	• Sphenoid wing<br>• Anterior midline<br>• Post fossa-midline<br>• Post fossa-lateral & posterior<br>• Tentorial<br>• Intraventricular<br>• Pineal region	
Location subcategory	Dropdown list/Check box<br><br>• Anterior<br>• Posterior<br>• Falco-tentorial<br>• Lateral<br>• Medial (including ACP)<br>• Cribriform plate/olfactory groove<br>• Planum<br>• Tuberculum/diaphragma sellae<br>• Clival<br>• Petro-clival<br>• Anterior foramen magnum<br>• Petrous<br>• Squamous occipital<br>• Posterior foramen magnum<br>• Supratentorial<br>• Infratentorial	Required entry. Allow one option only<br><br>Note to data collector: ICOM classification (appendix 2)<br><br>Appropriate subcategories will appear based on main category selected
Side	Dropdown list/check box<br><br>• Right<br>• Left<br>• Midline	Required entry. Allow one option only
**MANAGEMENT DECISION (PAGE/SECTION 4)**		

Decision	Dropdown list/Check box<br><br>• Active monitoring<br>• Surgery<br>• SRS<br>• *f*RT<br>• Discharge from outpatient care<br>• Lost to follow-up<br>• Dead	Required entry. Allow one option only<br><br>Note to data collector: You will be directed to the relevant page/section dependent on the choice. If the patient died or was lost to follow-up after this point in time, please choose one of the last two options, albeit the intention might have been to continue monitoring or intervene.
**ACTIVE MONITORING (SECTION/PAGE 5)**		
Scan date	DD/MM/YYYY	Required entry
Peritumoural signal intensity on T2	Dropdown list/check box<br><br>• 0-5%<br>• 6-33%<br>• 34-66%<br>• 67-100%<br>• NA	Required entry. Allow one option only<br><br>Note to data collector: In relation to tumour volume on T2/FLAIR MRI. If only CT or T1 MRI available, NA
Peritumoural signal intensity on FLAIR	Dropdown list/check box<br><br>• 0-5%<br>• 6-33%<br>• 34-66%<br>• 67-100%<br>• NA	Required entry. Allow one option only<br><br>Note to data collector: In relation to tumour volume on FLAIR MRI. If only CT or T1 MRI available, NA
Venous sinus nearby	Checkbox	Required entry.<br><br>Note to data collector: Tick box if within 10 mm, in direct contact or invading one of: superior sagittal sinus (SSS), sigmoid sinus (SS), transverse sinus (TS), cavernous sinus (CS) and the confluence of sinuses (CoS)
If yes, specify	Dropdown list/check box	Prompt entry if previous option (venous sinus nearby) has been ticked. Allow one option only

	• Superior sagittal sinus<br>• Cavernous sinus<br>• Sigmoid sinus<br>• Transverse sinus<br>• Confluence of sinuses	
Separate, direct contact or invaded?	Dropdown list/check box<br><br>• Separate<br>• Direct contact<br>• Invaded	Prompt entry if previous option (venous sinus nearby) has been ticked. Allow one option only
Any new meningioma-related symptoms?	Checkbox	Required entry
If yes, specify domain	Dropdown list/check box<br><br>• Seizure<br>• Headache<br>• Motor<br>• Sensory<br>• Language<br>• Cognitive<br>• Other	Prompt entry if previous option (Any new meningioma-related symptoms?) has been ticked. Allow multiple options
Major axis (mm)	Free field	Required entry<br><br>Note to data collector: In mm to 1 dp. Measure on axial T1/CT + GAD if available
Minor axis (mm)	Free field	Required entry<br><br>Note to data collector: In mm to 1 dp. Perpendicular to the major axis on axial T1/CT + GAD if available
Cor/sag major axis (mm)	Free field	Required entry<br><br>Note to data collector: In mm to 1 dp. Maximum height

Outcome	Dropdown list/check box<br><br>• Resume follow-up (active monitoring)<br>• Surgery<br>• SRS<br>• *f*RT<br>• Discharge<br>• Lost to follow-up<br>• Dead	Required entry. Allow one option only<br><br>Note to data collector: You will be directed to the relevant page/section dependent on the choice. If the patient died or was lost to follow-up after this interval scan, please choose one of the last two options, albeit the intention might have been to continue monitoring or intervene. If the option you choose is set to take place in the future, choose the option, save the data entered and exit the form with no further information required.
**SURGERY (SECTION/PAGE 6)**		
Surgery date	DD/MM/YYYY	Required entry
Indication for intervention	Dropdown list/checkbox<br><br>• Clinical-radiological<br>• Clinical<br>• Radiological<br>• Patient preference	Required entry. Allow one option only
Preoperative WHO PS	Dropdown list/checkbox<br><br>• 0<br>• 1<br>• 2<br>• 3<br>• 4	Required entry. Allow one option only
Preoperative comorbidities	Check box<br><br>• Hypertension - systolic > 140 or diastolic > 90 and patients on medical treatment<br>• Previous myocardial infarction<br>• Congestive heart failure	Required entry. Allow multiple options.<br><br>Note to data collector: Age and comorbidities will be used to calculate the age adjusted Charlson comorbidity index. Each of these is defined in the guide provided

	• Peripheral vascular disease<br>• Previous stroke/TIA - If hemiplegia present, do not check<br>• Hemi/paraplegia<br>• Diabetes which requires medical treatment<br>• Diabetes with end-organ damage - if so, do not check diabetes that requires treatment<br>• COPD/Asthma<br>• Renal disease<br>• Mild liver disease - Hep B/C or cirrhosis without portal hypertension<br>• Moderate to severe liver disease - cirrhosis with portal hypertension, jaundice, ascites<br>• Peptic ulcer disease<br>• Cancer - excluding basal cell carcinoma<br>• Metastatic cancer - if so, do not check cancer<br>• Rheumatic or connective tissue disease<br>• HIV/AIDS<br>• Skin ulcers/cellulitis<br>• Depression<br>• Dementia<br>• On Warfarin	
Simpson grade	Dropdown list/check box<br><br>• 1-GTR<br>• 2-GTR	Required entry. Allow one option only

	• 3-GTR<br>• 4-STR<br>• 5-STR	
WHO grade at the time of surgery	Dropdown list/check box<br><br>• 1<br>• 2<br>• 3	Required entry. Allow one option only<br><br>Note to data collector: According to the WHO classification at the time of surgery
Microscopic brain invasion	Dropdown list/checkbox<br><br>• Yes<br>• No<br>• Brain tissue absent<br>• NA	Required entry. Allow one option only<br><br>Note to data collector: as described in the pathology report.
Updated WHO grade (2016)	Dropdown list/check box<br><br>• 1<br>• 2<br>• 3<br>• NA	Required entry. Allow one option only. Only to appear if date of surgery <2017<br><br>Note to data collector: For meningiomas operated prior to 2016, grading will have been done according to the 2007 classification. The 2016 version can upgrade WHO grade 1 meningiomas to grade 2 if microscopic brain invasion is present. This means that WHO grade 2 and 3 meningiomas remain unchanged. Grade 1 meningiomas on the other hand can be upgraded in the presence of brain invasion. This requires review by a pathologist and so if not feasible, choose NA. For meningiomas classed according to the 2016 WHO classification, grade remains unchanged.
Postoperative surgical complications	Checkbox	Required
Complication	Dropdown list/check box<br><br>• Haemorrhage<br>• Hydrocephalus<br>• Surgical site infection - superficial and deep incisiona<br>• Surgical site infection - intracranial (meningitis, ventriculitis and abscess)	Prompt entry if previous option (Postoperative surgical complications) has been ticked<br><br>Allow more the one option

	• Stroke<br>• CSF leak<br>• Other	
New or worsening neurological impairment	Checkbox	Prompt entry if previous option (Postoperative surgical complications) has been ticked<br><br>Note to data collector: If symptoms present tick box. Note that some patients will have a radiological haemorrhage on postoperative imaging with no symptoms. Include these but don't tick clinical manifestation. On the other hand, some patients will have new symptoms such as seizure with no radiological cause, include these as well.
Clinical manifestation	Dropdown list/checkbox<br><br>• Seizure<br>• Headache<br>• Motor<br>• Sensory<br>• Language<br>• Cognitive<br>• Reduced GCS<br>• Other	Prompt entry if previous option (New or worsening neurological impairment) has been ticked. Allow multiple options
Pharmacological intervention	Checkbox	Prompt entry if previous option (Postoperative surgical complications) has been ticked
Surgical intervention	Dropdown list/checkbox<br><br>• No<br>• Without GA<br>• Under GA	Prompt entry if previous option (Postoperative surgical complications) has been ticked
ICU admission	Checkbox	Prompt entry if previous option (Postoperative surgical complications) has been ticked
Organ failure	Dropdown list/checkbox<br><br>• None<br>• Single-organ<br>• Multi-organ	Prompt entry if previous option (Postoperative surgical complications) has been ticked. Allow one option only

Persisted with no improvement beyond 30 days?	Checkbox	Prompt entry if previous option (Postoperative surgical complications) has been ticked
Postoperative medical complications	checkbox	Required
Complication	Dropdown list/check box<br><br>• Myocardial infarction<br>• Arrhythmia<br>• Pneumonia<br>• Pulmonary embolism<br>• Deep venous thrombosis<br>• Urinary tract infection<br>• Acute kidney injury<br>• Other	Prompt entry if previous option (Postoperative medical complications) has been ticked<br><br>Allow more than one option
Pharmacological intervention	Checkbox	Prompt entry if previous option (Postoperative medical complications) has been ticked
Surgical intervention	Dropdown list/checkbox<br><br>• No<br>• Without GA<br>• Under GA	Prompt entry if previous option (Postoperative medical complications) has been ticked
ICU admission	Checkbox	Prompt entry if previous option (Postoperative medical complications) has been ticked
Organ failure	Dropdown list/checkbox<br><br>• None<br>• Single-organ<br>• Multi-organ	Prompt entry if previous option (Postoperative medical complications) has been ticked. Allow one option only
Persisted with no improvement beyond 30 days?	Checkbox	Prompt entry if previous option (Postoperative medical complications) has been ticked
Postoperative WHO PS	Dropdown list/checkbox<br><br>• 0	Required. Allow one option only

	• 1<br>• 2<br>• 3<br>• 4<br>• 5 (dead)	
Recurrence	Checkbox	Prompt entry if previous option (Postoperative WHO PS) is not 5
Scan date (at recurrence or last follow-up date if no recurrence)	DD/MM/YYYY	Prompt entry if previous option (Postoperative WHO PS) is not 5
WHO PS at time of recurrence/last follow-up	Dropdown list/checkbox<br>• 0<br>• 1<br>• 2<br>• 3<br>• 4	Prompt entry if previous option (Postoperative WHO PS) is not 5
**SRS (SECTION/PAGE 7)**		
Pre-radiation WHO PS	Dropdown list/check box<br>• 0<br>• 1<br>• 2<br>• 3<br>• 4	Required entry. Allow one option only
Pre-radiation comorbidity	Check box<br>• Hypertension - systolic > 140 or diastolic > 90 and patients on medical treatment<br>• Previous myocardial infarction<br>• Congestive heart failure<br>• Peripheral vascular disease	Required entry. Allow multiple options.<br>Note to data collector: Age and comorbidities will be used to calculate the age adjusted Charlson comorbidity index. Each of these is defined in the guide provided

	• Previous stroke/TIA - If hemiplegia present, do not check<br>• Hemi/paraplegia<br>• Diabetes which requires medical treatment<br>• Diabetes with end-organ damage - if so, do not check diabetes that requires treatment<br>• COPD/Asthma<br>• Renal disease<br>• Mild liver disease - Hep B/C or cirrhosis without portal hypertension<br>• Moderate to severe liver disease - cirrhosis with portal hypertension, jaundice, ascites<br>• Peptic ulcer disease<br>• Cancer - excluding basal cell carcinoma<br>• Metastatic cancer - if so, do not check cancer<br>• Rheumatic or connective tissue disease<br>• HIV/AIDS<br>• Skin ulcers/cellulitis<br>• Depression<br>• Dementia<br>• On Warfarin	
Dose	Free field	Required entry
Early CTCAE toxicity (≤3 months)	Checkbox	Required entry

Toxicity	Free field	Prompt entry if previous option (Early CTCAE toxicity (≤3 months)) is ticked<br><br>Note to data collector: https://ctep.cancer.gov/protocoldevelopment/electronic_applications/ctc.htm<br><br>Use the following format: System class organ (SOC)-CTCAE term-grade e.g. nervous system disorders-headache-1. Select the complication with the highest toxicity grade
Late CTCAE toxicity	Checkbox	Required entry
Toxicity	Free field	Prompt entry if previous option (Late CTCAE toxicity) is ticked<br><br>Note to data collector: https://ctep.cancer.gov/protocoldevelopment/electronic_applications/ctc.htm<br><br>Use the following format: System class organ (SOC)-CTCAE term-grade e.g. nervous system disorders-headache-1. Select the complication with the highest toxicity grade
Meningioma progression/regrowth	Checkbox	Required
Scan date (at progression or last follow-up date if no progression)	DD/MM/YYYY	Required
WHO PS at time of progression/last follow-up	Dropdown list/checkbox<br><br>• 0<br>• 1<br>• 2<br>• 3<br>• 4<br>• 5 (dead)	Required. Allow one option only
***f*RT (SECTION/PAGE 8)**		

Pre-radiation WHO PS	Dropdown list/check box<br><br>• 0<br>• 1<br>• 2<br>• 3<br>• 4	Required entry
Pre-radiation comorbidity	Check box<br><br>• Myocardial infarction<br>• Congestive heart failure<br>• Peripheral vascular disease<br>• Hemiplegia<br>• Cerebrovascular disease<br>• Pulmonary disease<br>• Diabetes<br>• Renal disease<br>• Liver disease<br>• Peptic ulcer disease<br>• Cancer<br>• Dementia<br>• Connective tissue disease<br>• AIDS<br>• Hypertension<br>• Skin ulcers/cellulitis<br>• Depression<br>• On Warfarin	Required entry. Allow multiple options.<br><br>Note to data collector: Age and comorbidities will be used to calculate the age adjusted Charlson comorbidity index. Each of these is defined in the guide provided
Number of fractions	Free field	Required entry
Fractionated dose	Free field	Required entry
Total dose	Free field	Required entry
Early CTCAE toxicity (≤3 months)	Checkbox	Required entry

Toxicity	Free field	Prompt entry if previous option (Early CTCAE toxicity (≤3 months)) is ticked<br><br>Note to data collector: https://ctep.cancer.gov/protocoldevelopment/electronic_applications/ctc.htm<br><br>Use the following format: System class organ (SOC)-CTCAE term-grade e.g. nervous system disorders-headache-1. Select the complication with the highest toxicity grade
Late CTCAE toxicity	Checkbox	Required entry
Toxicity	Free field	Prompt entry if previous option (Late CTCAE toxicity) is ticked<br><br>Note to data collector: https://ctep.cancer.gov/protocoldevelopment/electronic_applications/ctc.htm<br><br>Use the following format: System class organ (SOC)-CTCAE term-grade e.g. nervous system disorders-headache-1. Select the complication with the highest toxicity grade
Meningioma progression/regrowth	Checkbox	Required
Scan date (at progression or last follow-up date if no progression)	DD/MM/YYYY	Required
WHO PS at time of progression/last follow-up	Dropdown list/checkbox<br><br>• 0<br>• 1<br>• 2<br>• 3<br>• 4<br>• 5 (dead)	Required. Allow one option only
**Discharge from outpatient care/Lost to follow-up (SECTION/PAGE 9)**		

Date of data entry into the database	DD/MM/YYYY	Required entry
Rescanned during the time between discharge/loss to FU and the date of data entry	Checkbox	Required entry
Date of scan	DD/MM/YYYY	Prompt entry if previous option (Readmitted/rescanned during the time between discharge/loss to FU and the date of data entry) has been checked
Reason?	Dropdown list:<br>• Seizure<br>• Headache<br>• Motor<br>• Sensory<br>• Language<br>• Cognitive<br>• Other	Prompt entry if previous option (Readmitted/rescanned during the time between discharge/loss to FU and the date of data entry) has been checked. Allow multiple options
Peritumoural signal intensity on T2	Dropdown list/check box<br>• 0-5%<br>• 6-33%<br>• 34-66%<br>• 67-100%<br>• NA	Prompt entry if previous option (Readmitted/rescanned during the time between discharge/loss to FU and the date of data entry) has been checked. Allow one option only<br>Note to data collector: In relation to tumour volume. If only baseline CT available, NA
Peritumoural signal intensity on FLAIR	Dropdown list:<br>• 0-5%<br>• 6-33%<br>• 34-66%<br>• 67-100%<br>• NA	Prompt entry if previous option (Readmitted/rescanned during the time between discharge/loss to FU and the date of data entry) has been checked. Allow one option only<br>Note to data collector: In relation to tumour volume. If only baseline CT available, NA
Venous sinus nearby	Checkbox<br>• Superior sagittal sinus<br>• Cavernous sinus	Prompt entry if previous option (Readmitted/rescanned during the time between discharge/loss to FU and the date of data entry) has been checked

	• Sigmoid sinus<br>• Transverse sinus<br>• Confluence of sinuses	Note to data collector: Tick box if within 10 mm, in direct contact or invading one of: superior sagittal sinus (SSS), sigmoid sinus (SS), transverse sinus (TS), cavernous sinus (CS) and the confluence of sinuses (CoS)
If yes, specify	Dropdown list/check box	Prompt entry if previous option (venous sinus nearby) has been ticked. Allow one option only
Separate, direct contact or invaded?	Dropdown list/check box<br><br>• Separate<br>• Direct contact<br>• Invaded	Prompt entry if previous option (venous sinus nearby) has been ticked. Allow one option only
Major axis (mm)	Free field	Prompt entry if previous option (Readmitted/rescanned during the time between discharge/loss to FU and the date of data entry) has been checked<br><br>Note to data collector: In mm to 1 dp. Measure on axial T1/CT + GAD
Minor axis (mm)	Free field	Prompt entry if previous option (Readmitted/rescanned during the time between discharge/loss to FU and the date of data entry) has been checked<br><br>Note to data collector: In mm to 1 dp. Perpendicular to the major axis on axial T1/CT + GAD
Cor/sag major axis (mm)	Free field	Prompt entry if previous option (Readmitted/rescanned during the time between discharge/loss to FU and the date of data entry) has been checked<br><br>Note to data collector: In mm to 1 dp. Maximum height
Verdict	Dropdown list/checkbox<br><br>• Related<br>• Unrelated	Prompt entry if previous option (Readmitted/rescanned during the time between discharge/loss to FU and the date of data entry) has been checked. Allow one option only<br><br>Note to data collector: Were the symptoms attributed to the meningioma?
Outcome	Dropdown list/Checkbox	Prompt entry if previous option (Readmitted/rescanned during the time between discharge/loss to FU and the date of data entry) has been checked. Allow one option only

	• Resume follow-up (active monitoring)<br>• Surgery<br>• SRS<br>• *f*RT<br>• Discharge<br>• Lost to follow-up<br>• Dead	Note to data collector: You will be directed to the relevant page/section dependent on the choice. If the patient died or was lost to follow-up after this interval scan, please choose one of the last two options, albeit the intention might have been to continue monitoring or intervene. If the option you choose is set to take place in the future, choose the option, save the data entered and exit the form with no further information required.
Overall outcome	Dropdown list/Checkbox:<br><br>• Dead<br>• Alive	Prompt entry if previous option (Rescanned during the time between discharge/loss to FU and the date of data entry) is not ticked.<br><br>Allow one option only<br><br>Note to data collector: You will be directed to section 10 if Dead is selected.
**Mortality (SECTION/PAGE 10)**		
Date of death	DD/MM/YYYY	Required entry
Cause of death	Dropdown list/checkbox<br><br>• Meningioma-related<br>• Unrelated	Required entry. Allow one option only<br><br>Note to data collector: An example of a meningioma-related death would be for example status epilepticus in a patient who manifested seizures but didn't have treatment. An unrelated death would be for example a community acquired pneumonia. For the purpose of the study, any death occurring from a morbidity, which did not necessitate neurosurgical input/opinion will be classified as unrelated

1.2. Appendix. 2. ICOM classification of meningioma location

ICOM classification system of meningioma locations			
**Main category**	Subcategories		
**Convexity**	Anterior [1]	Posterior[1]	
**Parasagittal**	Anterior[1]	Posterior[1]	Falco-tentorial
**Parafalcine**	Anterior[1]	Posterior[1]	Falco-tentorial
**Sphenoid wing**	Lateral	Medial (including ACP)	
**Anterior midline**	Cribriform plate or olfactory groove[2]	Planum[3]	Tuberculum and diaphragma sellae
**Posterior fossa - midline**	Clival	Petro-clival	Anterior foramen magnum[4]
**Posterior fossa – Lateral & posterior**	Petrous	Squamous occipital	Posterior foramen magnum[4]
**Tentorial**	Supratentorial	Infratentorial	
**Intraventricular**			
**Pineal region[5]**			

[1] The main attachment is located anterior or posterior, respectively, to the coronal suture
[2] Arising between the crista galli and the fronto-sphenoid suture
[3] Arising between the fronto-sphenoid suture and the limbus sphenoidale
[4] The main attachment is located anterior or posterior, respectively, to the hypoglossal canal
[5] No obvious tentorial attachment
