# Supplementary material for: External validation and recalibration of an incidental meningioma prognostic model – IMPACT: protocol for an international multicentre retrospective cohort study
Source: BMJ Open. 2022 Jan 18;12(1):e052705. doi: 10.1136/bmjopen-2021-052705 (PMC8768908; doi:10.1136/bmjopen-2021-052705)
Supplement: Author's manuscript [file bmjopen-2021-052705.draft_revisions.pdf]

BMJ Open

BMJ Open is committed to open peer review. As part of this commitment we make the peer review history of every article we publish publicly available.

When an article is published we post the peer reviewers’ comments and the authors’ responses online. We also post the versions of the paper that were used during peer review. These are the versions that the peer review comments apply to.

The versions of the paper that follow are the versions that were submitted during the peer review process. They are not the versions of record or the final published versions. They should not be cited or distributed as the published version of this manuscript.

BMJ Open is an open access journal and the full, final, typeset and author-corrected version of record of the manuscript is available on our site with no access controls, subscription charges or pay-per-view fees (http://bmjopen.bmj.com).

If you have any questions on BMJ Open’s open peer review process please email info.bmjopen@bmj.com

BMJ Open

External validation and recalibration of an incidental meningioma prognostic model – IMPACT: protocol for an international multicentre retrospective cohort study

Journal:	*BMJ Open*
Manuscript ID	bmjopen-2021-052705
Article Type:	Protocol
Date Submitted by the Author:	25-Apr-2021
Complete List of Authors:	Islim, Abdurrahman ; University of Liverpool, Institute of Systems, Molecular, & Integrative Biology; The Walton Centre NHS Foundation Trust, Department of Neurosurgery<br>millward, Christopher; University of Liverpool, Institute of Systems, Molecular, & Integrative Biology; The Walton Centre NHS Foundation Trust, Department of Neurosurgery<br>Piper, Rory; John Radcliffe Hospital, Department of Neurosurgery<br>Fountain, Daniel; Salford Royal NHS Foundation Trust, Manchester Centre for Clinical Neurosciences<br>Mehta, Shaveta; Clatterbridge Cancer Centre NHS Foundation Trust, Department of Clinical Oncology; University of Liverpool, Institute of Systems, Molecular, & Integrative Biology<br>Kolamunnage-Dona, Ruwanthi; University of Liverpool, Department of Health Data Science, Institute of Population Health<br>Ali, Usama; University of Oxford, Clinical Trial Service Unit and Epidemiological Studies Unit, Nuffield Department of Population Health<br>Koszdin, Shelli; Veterans Affairs Healthcare System<br>Georgious, Theo; Brain Tumor Charity<br>Mills, Samantha; The Walton Centre NHS Foundation Trust, Department of Neuroradiology<br>Brodbelt, Andrew; University of Liverpool, Institute of Systems, Molecular, & Integrative Biology; The Walton Centre NHS Foundation Trust, Department of Neurosurgery<br>Mathew, Ryan; University of Leeds School of Medicine, Leeds Institute of Medical Research at St James's; Leeds General Infirmary, Department of Neurosurgery<br>Santarius, Thomas; Addenbrooke's Hospital, Department of Neurosurgery; University of Cambridge School of Clinical Medicine, Division of Neurosurgery<br>Jenkinson, Michael; University of Liverpool, Institute of Systems, Molecular, & Integrative Biology; The Walton Centre NHS Foundation Trust, Department of Neurosurgery
Keywords:	Neurological oncology < ONCOLOGY, NEUROSURGERY, Neuroradiology < NEUROLOGY

SCHOLARONE™
Manuscripts

I, the Submitting Author has the right to grant and does grant on behalf of all authors of the Work (as defined in the below author licence), an exclusive licence and/or a non-exclusive licence for contributions from authors who are: i) UK Crown employees; ii) where BMJ has agreed a CC-BY licence shall apply, and/or iii) in accordance with the terms applicable for US Federal Government officers or employees acting as part of their official duties; on a worldwide, perpetual, irrevocable, royalty-free basis to BMJ Publishing Group Ltd ("BMJ") its licensees and where the relevant Journal is co-owned by BMJ to the co-owners of the Journal, to publish the Work in this journal and any other BMJ products and to exploit all rights, as set out in our licence.

The Submitting Author accepts and understands that any supply made under these terms is made by BMJ to the Submitting Author unless you are acting as an employee on behalf of your employer or a postgraduate student of an affiliated institution which is paying any applicable article publishing charge ("APC") for Open Access articles. Where the Submitting Author wishes to make the Work available on an Open Access basis (and intends to pay the relevant APC), the terms of reuse of such Open Access shall be governed by a Creative Commons licence – details of these licences and which Creative Commons licence will apply to this Work are set out in our licence referred to above.

Other than as permitted in any relevant BMJ Author's Self Archiving Policies, I confirm this Work has not been accepted for publication elsewhere, is not being considered for publication elsewhere and does not duplicate material already published. I confirm all authors consent to publication of this Work and authorise the granting of this licence.

For peer review only

External validation and recalibration of an incidental meningioma prognostic model – IMPACT: protocol for an international multicentre retrospective cohort study

Author and affiliations:

Abdurrahman I. Islim[1,2*], Christopher P. Millward[1,2], Rory J. Piper[3], Daniel M Fountain[4], Shaveta Mehta[1,5], Ruwanthi Kolamunnage-Dona[6], Usama Ali[7], Shelli Diane Koszdin[8], Theo Georgious[9], Samantha J. Mills[10], Andrew R. Brodbelt[1,2], Ryan K. Mathew[11,12], Thomas Santarius[13,14], Michael D. Jenkinson[1,2*] on behalf of the IMPACT Study Investigators, International Consortium on Meningioma (ICOM) and British Neurosurgical Trainee Research Collaborative (BNTRC)

1. Institute of Systems, Molecular, & Integrative Biology, University of Liverpool, Liverpool, UK
2. Department of Neurosurgery, The Walton Centre NHS Foundation Trust, Liverpool, UK
3. Department of Neurosurgery, John Radcliffe Hospital, Oxford University Hospitals NHS Foundation Trust, Oxford, UK
4. Manchester Centre for Clinical Neurosciences, Salford Royal NHS Foundation Trust, Salford, UK
5. Department of Clinical Oncology, The Clatterbridge Cancer Centre NHS Foundation Trust, Wirral, UK
6. Department of Health Data Science, Institute of Population Health, University of Liverpool, Liverpool, UK
7. Clinical Trial Service Unit and Epidemiological Studies Unit, Nuffield Department of Population Health, University of Oxford, Oxford, UK
8. Veterans Affairs Healthcare System, Palo Alto, California, USA
9. The Brain Tumour Charity, Fleet, Hampshire, UK
10. Department of Neuroradiology, The Walton Centre NHS Foundation Trust, Liverpool, UK
11. Leeds Institute of Medical Research at St James's, University of Leeds, Leeds, UK
12. Department of Neurosurgery, Leeds Teaching Hospitals NHS Trust, Leeds, UK
13. Department of Neurosurgery, Addenbrooke's Hospital, Cambridge, UK
14. Division of Neurosurgery, School of Clinical Medicine, University of Cambridge, Cambridge, UK

***Corresponding Authors**

Dr Abdurrahman I Islim, MPhil and Professor Michael D Jenkinson, FRCS, PhD
Institute of Systems, Molecular, & Integrative Biology, University of Liverpool
Department of Neurosurgery, The Walton Centre NHS Foundation Trust
Lower Lane
Liverpool, L9 7LJ
Tel: +44 (0)151 795 5996
Emails: a.islim@doctors.org.uk and Michael.Jenkinson@liverpool.ac.uk

Word count:

Abstract, 355 words; Manuscript, 3613 words; References, 699 words

For peer review only - http://bmjopen.bmj.com/site/about/guidelines.xhtml

ABSTRACT

Introduction: Due to the increased use of computed tomography and magnetic resonance imaging, the prevalence of incidental findings on brain scans is increasing. Meningioma, the commonest primary brain tumour, is a frequently encountered incidental finding, with an estimated prevalence of 3/1000. The management of incidental meningioma varies widely with active clinical-radiological monitoring being the most accepted method by clinicians. Duration of monitoring and time intervals for assessment, however, are not well defined. To this end, we have recently developed a statistical model of progression risk based on single-centre retrospective data. The model – IMPACT (***I***ncidental ***M***eningioma: ***P***rognostic ***A***nalysis Using Patient ***C***omorbidity and MRI ***T***ests) employs baseline clinical and imaging features to categorise the patient with an incidental meningioma into one of three risk groups: low-, medium- and high-risk with a proposed active monitoring strategy based on the risk and temporal trajectory of progression, accounting for actuarial life expectancy. The primary aim of this study is to assess the external validity of this model.

Methods and analysis: IMPACT is a retrospective multi-centre study which will aim to include 1500 patients with an incidental intracranial meningioma, powered to detect a 10% progression risk. Adult patients ≥16 years diagnosed with an incidental meningioma between 01/01/2009 and 31/12/2010 will be included. Clinical and radiological data will be collected longitudinally until the patient reaches one of the study endpoints: intervention (surgery, stereotactic radiosurgery, or fractionated radiotherapy), mortality or last date of follow-up. Data will be uploaded to an online REDCap database with no unique identifiers. External validity of IMPACT will be tested using established statistical methods.

Ethics and dissemination: Local institutional approval at each participating centre will be required. Results of the study will be reported through peer-reviewed articles and conferences and disseminated to participating centres, patients and the public using social media.

Registration details: Researchregistry6051

For peer review only

STRENGTHS AND LIMITATIONS

- The first multi-centre international study to investigate the prognosis of incidental intracranial meningiomas
- The study will include a large cohort of 1500 patients
- The longitudinal study design with serial collection of clinical and imaging data will provide a unique insight into meningioma behaviour and provide a platform for future investigation of novel biomarkers
- The retrospective nature of the study may bias patient and information selection
- Results of the study may be biased by clinician and patient management preference in each participating centre

For peer review only

For peer review only - http://bmjopen.bmj.com/site/about/guidelines.xhtml

INTRODUCTION

Meningiomas have the highest incidence rate amongst all primary central nervous system tumours. Descriptive studies from Europe and North America suggest this rate is between 4.20 and 8.58 per 100,000 individuals (1, 2). Wider access and increased use of magnetic resonance imaging (MRI) and computed tomography (CT) has led to a marked rise in the number of incidental findings in clinical and research settings. Meningiomas comprise 15% of incidental findings on brain MRI and have a prevalence of 3 per 1000 (3). A recent study of the Surveillance, Epidemiology, and End Results (SEER) database demonstrated a substantial increase in the detection of smaller, incidental tumours; between 2004 and 2012, the proportion of meningiomas <1 cm in diameter, diagnosed in a given year, increased in a linear fashion from 6 to 11% (4). Incidental, asymptomatic meningiomas cause patient anxiety and uncertainly around the need for future treatment and often prompt clinicians to commence long-term MRI and clinical follow-up. International consensus guidelines by the European Association of Neuro-Oncology (EANO) and National Comprehensive Cancer Network (NCCN) suggest active monitoring with MRI as first line for managing these tumours (5, 6), but data to advise on the optimal follow-up duration and screening intervals is currently lacking (7).

Previous studies have identified prognostic radiological factors that are associated with the risk of meningioma growth and development of clinical symptoms; yet the timing of such progression is poorly defined (8-10). Moreover, clinical factors such as patient comorbidity and performance status remain unexplored in relation to prognosis but are highly relevant. The patient with an incidental meningioma wants to know whether their tumour will grow and become symptomatic such that it will require safe treatment within their healthy lifetime.

To this end, a recent retrospective cohort study of incidental meningioma patients in the United Kingdom (UK) was conducted to assess the utility of combining routinely available radiological and clinical factors to develop a prognostic model for the risk of incidental meningioma progression during active monitoring (11). The model IMPACT (***I***ncidental ***M***eningioma: ***P***rognostic ***A***nalysis Using Patient ***C***omorbidity and MRI ***T***ests) could be used as a tool to guide active monitoring strategies for patients with an incidental asymptomatic meningioma within the first 10 years of diagnosis, however validation with external datasets is required.

For peer review only - http://bmjopen.bmj.com/site/about/guidelines.xhtml

The primary aim of this international retrospective cohort study of incidental meningioma is to externally validate and calibrate the prognostic model IMPACT, accessible using https://www.impact-meningioma.com. These data will provide insight into the incidence, epidemiology, presentation, management, and long-term outcomes of incidental meningioma, which will inform the development of clinical guidelines and identify areas for future research.

THE IMPACT MODEL

The model, based on MRI parameters, stratifies patients with an incidental meningioma into three risk groups: low-, medium- and high-risk. These MRI parameters are as follows: meningioma volume, meningioma hyperintensity, peritumoral signal change and proximity to critical neurovascular structures. This predictive function was built using an internally validated cox regression model. Patients were also stratified in the model based on age, comorbidity and performance status using competing risk analyses.

OBJECTIVES

Primary objective

To externally validate the prognostic model IMPACT

Secondary objectives

- To update the parameters of the prognostic model IMPACT if measures of external validation demonstrate a poor fit, and internally validate the updated model
- To determine the growth patterns of incidental meningiomas
- To examine the MRI and pathology features of meningiomas subject to surgical resection
- To determine the risk of post-intervention complications and tumour recurrence/growth for meningiomas subject to surgery, stereotactic radiosurgery, or fractionated radiotherapy
- Assess the economic implications of stratifying follow-up according to risk of disease progression

For peer review only - http://bmjopen.bmj.com/site/about/guidelines.xhtml

METHODS AND ANALYSIS

Study design

This will be a retrospective, international multicentre cohort study. The study will include incidental meningioma patients managed at each participating centre. Cases will be identified by the local site research teams using existing patient medical records. Baseline clinical and radiological characteristics, tumour management, and clinical and radiological outcomes will be collected and recorded (anonymised data) on a secure database by the local investigator. Since this study falls within the remit of clinical outcomes audit, individual patient consent is not required. The study will collect data from the medical records for patients newly-diagnosed over a 2-year period between 1st January 2009 and 31st December 2010. This is an observational study and will not alter routine patient care.

Study population and eligibility criteria

The study will include adults (≥16 years of age) with a newly identified incidental intracranial meningioma, as per radiology report, diagnosed between 1st January 2009 and 31st December 2010. Radiological diagnosis is expected to be based on the presence of an extra-axial lesion with broad-based attachment along the dura showing contrast enhancement. The accepted definition of an incidental finding is “a previously undetected abnormality of potential clinical relevance that is unexpectedly discovered and unrelated to the purpose of the examination”.

Exclusion criteria are as follows:

- History of cranial radiation >5 years before diagnosis
- History of neurofibromatosis type 2
- Surgical resection which revealed a different histopathological diagnosis
- Unavailability of medical notes

Patient identification

Eligible patients can be identified using local radiology information systems, for example the Computerised Radiological Information System (CRIS) tool. The search strategy will involve

For peer review only - http://bmjopen.bmj.com/site/about/guidelines.xhtml

review of the medical records of all patients managed with a meningioma at participating centres and exclusion of those that do not meet the selection criteria (Figure 1).

Sample size

For external validation studies, a minimum of 100 events is required (12). The risk of incidental meningioma progression is estimated to be 10% (11). Based on this, data for 1000 patients will be required. To account for variability in the progression risk, follow-up regimes and loss to follow-up, we will aim to include a minimum of 1500 patients across participating centres. An interim analysis will be conducted after data for 500 patients have been collected to assess for the risk of incidental meningioma progression and review the required number of patients.

Study endpoints

Primary endpoint

Disease progression will be defined using a composite endpoint comprising of new symptom development, meningioma-specific mortality, meningioma growth (absolute growth rate ≥2 cm^3/year or absolute growth rate≥1 cm^3/year + relative growth rate ≥30%/year), development or increase of peritumoural brain oedema (defined as increased signal intensity on T2/FLAIR), venous sinus invasion and meningioma volume exceeding 10 cm^3. The first two criteria denote clinical progression while the latter three are related to loss of window of curability. Venous sinus invasion and peritumoural oedema can prevent complete surgical resection (13, 14). Peritumoural oedema and a meningioma volume >10 cm^3 are relative contraindications to stereotactic radiosurgery (15, 16).

Secondary endpoints

Intervention (surgery, stereotactic radiosurgery, or fractionated radiotherapy) and mortality unrelated to the meningioma.

For peer review only - http://bmjopen.bmj.com/site/about/guidelines.xhtml

Data collection

Data will be collected at each centre by members of the local team. Data will be collected from the patient's medical and radiology records. All clinical and radiological information collected for this study by the local investigators should be available routinely and no extra patient assessment will be required. Data will be collected and stored online through a secure University of Liverpool server running the Research Electronic Data Capture (REDCap) web application and using the patient unique study number. Local investigators will be given secure REDCap project server login details. No patient identifiable information will be uploaded or stored on the REDCap database. The study number (site ID_patient ID) is generated by REDCap on creating a new patient record in the database. The clinical team can only view the records of patients from their own centre. All local investigators will store a copy of the link between the patient's unique study number and their patient identifiers on a secure password protected computer, using a blank link file provided by the study team.

REDCap database

REDCap is a secure web application for building and managing online databases. Access to REDCap will be provided by the Liverpool Clinical Trials Centre (LCTC), University of Liverpool, a partner of the REDCap consortium. Database programmers will oversee the development of a data collection tool (Appendix 1) which can be accessed using any electronic device with internet access. The database will be built to comply with the UK's Data Protection Act 2018 and the European Union's General Data Protection Regulation (GDPR). Quality assessment of the tool will be done over two phases. Phase 1 will involve local testing of the tool using pre-existing data (11). Phase 2 will expand testing to three to five additional participating centres. After completion of phase 2, the data collection tool will be made live for use by the participating sites.

For peer review only

Recorded variables

Baseline clinical variables

Age at diagnosis, sex, ethnicity, the World Health Organisation (WHO) performance status (PS) and the age adjusted Charlson comorbidity index (ACCI) (Table 1) (17-19). These factors will only be recorded at baseline.

Baseline radiological variables

Baseline imaging variables assessed will be:

- Single or multiple intracranial meningioma
- Tumour signal intensity compared to the contralateral grey matter on fluid attenuated inversion recovery (FLAIR) and T2-weighted (T2) MRI (hypo/iso/hyper) (Figure 2)
- Peritumoural signal intensity in relation to tumour volume using the signal change present on FLAIR and T2 MRI (0-5%/6-33%/34-66%/67-100%; adapted from the VASARI [Visually AcceSAble Rembrandt Images] MR features for gliomas (20))
- Meningioma volume using the ABC/2 formula on contrast-enhanced T1-weighted MRI/CT: (A) maximum meningioma diameter on axial plane, (B) diameter perpendicular to (A) and (C) maximum height on coronal/sagittal plane, not taking into the account the dural tail
- Meningioma location classed into non-skull base and skull base and further subcategorised according to the ICOM (International Consortium on Meningioma) classification system (Appendix 2)
- Proximity to major dural venous sinuses (superior sagittal sinus/transverse sinus/sigmoid sinus/cavernous sinus/the confluence of sinuses) categorised as separate (within 10 mm), in direct contact with its wall, or invading, excluding the dural tail (Figure 2)
- Contact with critical neurovascular structures (i.e. internal carotid artery and optic apparatus)

Meningiomas that fulfil one of the two previous categories are said to be in proximity to critical neurovascular structures. A video manual prepared by the study team will be made available to assist with standardisation and quality assurance of scan interpretation across participating centres.

For peer review only - http://bmjopen.bmj.com/site/about/guidelines.xhtml

Management strategy

Management strategies will include active monitoring, intervention (surgery, stereotactic radiosurgery [SRS] and fractionated radiotherapy [*f*RT]) or discharge from outpatient clinic care (Figure 3). **Active monitoring** is defined as regular surveillance imaging and outpatient clinical observation. Recorded factors will include:

- Number of scans, and interval between them (months)
- For each scan: peritumoural signal intensity, venous sinus involvement and meningioma volume
- Each scan will be examined alongside its corresponding outpatient clinic appointment for any evidence of meningioma-related symptoms (motor/sensory/language/cognitive/seizure/headache/other)
- The outcome of each clinical encounter (i.e. outpatient appointment) will be recorded (resume follow-up/surgery/SRS/*f*RT/hospital discharge)

Intervention details; if performed, will also be recorded. These will include indication for intervention (clinical-radiological/clinical/radiological/patient preference) and time to intervention. For patients treated with clinical-radiological or clinical progression, status of meningioma-related neurological morbidity will be noted.

For surgery, the following will additionally be recorded:

- Simpson grade (as recorded by the surgeon in the operative notes) (21)
- WHO grade (classified according to the WHO system in use at the time of surgery and updated according to the WHO 2016 classification dependent on pathologists' availability (22)) and presence of any reported brain invasion (yes / no / not reported)
- Postoperative medical and surgical complications recorded at 30 days (Landriel-Ibañez Classification (Table 2) (23)).
- Follow-up duration (months)
- WHO performance status pre- and postoperatively and at the last follow-up appointment.
- Recurrence on contrast-enhanced MRI during that time (yes/no) and if recurred then the time to recurrence

For SRS and *f*RT:

For peer review only - http://bmjopen.bmj.com/site/about/guidelines.xhtml

- Fractionated dose (*f*RT), number of fractions (*f*RT) and total dose (*f*RT /SRS)
- Early and late (≥3 months) toxicity (assessed by CTCAE v5.0, https://ctep.cancer.gov/protocoldevelopment/electronic_applications/ctc.htm).
- Duration of follow-up post-radiation (months)
- WHO performance status pre- and post-radiation and at the last follow-up appointment
- Progression/regrowth on contrast-enhanced MRI during that time (yes/no) and if progressed/regrew then the time to progression/regrowth

For patients discharged from outpatient care, data sources will be checked for any readmissions/rescans thought to be attributed to the incidental meningioma within the study timeframe; date of diagnosis up to the date of data entry. Outcome following readmissions/rescans will be noted.

Overall outcomes

Overall outcomes by the end of the study period (discharge from outpatient care/lost to follow-up/dead/under on-going active follow-up) and follow-up durations will be recorded. Any deaths encountered during follow-up will be recorded. The medical records for patients who are discharged will also be examined for mortality data.

Data quality assurance

An e-learning module will be prepared by the study team. This will contain the data collection guides and video manual. Upon completion, each study investigator will need to undergo a five-item assessment. An iterative process in which investigators have to redo the assessment or module will dictate their progress as follows:

- An assessment percentage of 100% will indicate successful completion of the module, which will allow the investigator to collect data for the study
- An assessment percentage less 100% will require repeating the assessment
- Five attempts will be allowed
- Subsequent failed attempts will entail review of the module components again

For peer review only - http://bmjopen.bmj.com/site/about/guidelines.xhtml

Planned statistical analysis

Demographic differences across groups will be explored with the χ2 test for categorical variable and the Mann-Whitney U test or Student's t-test for continuous variables. Correlation between baseline variables will be evaluated using the Pearson correlation coefficient. Normally distributed continuous variables will be expressed as mean (standard deviation [SD]) whereas skewed variables as median (interquartile range [IQR]). Differences will be considered statistically significant at $P<0.05$.

External validation

Using IMPACT, the five- and ten-year estimated risk of disease progression for every patient included in this cohort study will be calculated. Kaplan-Meier method will be used to obtain the observed risks. The predictive performance of IMPACT will be assessed by examining measures of calibration and discrimination. Calibration refers to how closely the predicted 5- and 10-year risk of progression agrees with the observed risk. A calibration plot compares the observed and predicted rates of events for each group. A perfect match indicates accurate calibration. The Brier score for censored survival data will also be calculated, which is a measure of accuracy and is the average squared deviation between predicted and observed risk; a lower score represents greater accuracy. Discrimination is the ability of the risk score to differentiate between those patients who do and those who do not experience disease progression during the study timeframe. This measure is quantified by calculating the C-statistic, D-statistic and Chambless and Diao's time-dependent AUC (area under the receiver operating characteristic curve [ROC]) which are tailored towards censored survival data. The proportional hazards assumption of the model will be tested by examination of Schoenfeld residuals, and influential observations will be examined using DFBETA panels.

The two competing risk analyses performed to build the IMPACT model will be repeated with the external dataset and plots of cumulative incidence rate (CIR) and 95% confidence intervals (CIs) will be compared to the original cohort (11). Patients will be split based on WHO PS into two categories: 0-1 and 2-4 and stratified by ACCI (Table 1) into three groups: 0-2, 3-5 and ≥6. The first analysis will assess the CIR of primary intervention at different time points following diagnosis stratified by PS and ACCI groups and the second analysis will evaluate the CIR of disease progression. The competing event for the former will be non-meningioma-specific mortality either observed during follow-up or after being discharged from outpatient care.

For peer review only - http://bmjopen.bmj.com/site/about/guidelines.xhtml

Patients who remain under follow-up will be censored at the last outpatient clinic appointment. Patients discharged alive from outpatient care will censored at the last time they were seen by a healthcare physician up to the date of data entry. For the disease progression analysis, four events will be considered competing in nature, discharge from outpatient care, loss to follow-up, death during follow-up or an intervention before disease progression occurred with the first three grouped together. Censoring will only be done for patients who remain under follow-up at the last clinic appointment. To test the equality across CIR groups, the Fine and Gray test will be carried out.

Model recalibration

If calibration and discrimination measures of external validation demonstrate a poor fit, the model will be recalibrated and adjusted using the data of included patients. This will be done over four stages:

- Stage 1: The regression coefficients will be recalibrated. This will be done using a Cox regression model fitted with the linear predictor as the only covariate
- Stage 2: The recalibrated model predictors will each be removed in a stepwise manner by a non-automated criterion-based procedure starting with the variable with a hazard ratio closest to 1. After removal of this variable, the aforementioned measures of discrimination of calibration and discrimination will be reassessed to detect model improvement. If the performance of the model is unimproved or worsens, the variable will be reintroduced to the model. This step will be repeated in a staged manner until no further improvements are detected. Introduction of new predictive variables will be possible
- Stage 3: The internal validity of the updated model will be assessed using a bootstrapping method
- Stage 4: Adjusted stratification by ACCI and PS (Table 1) will be performed to achieve statistically significant differences in equality across cumulative incidence rate groups, judged by the Fine and Gray test

Additional analyses

We envisage that imaging protocols in the participating centres are varied and non-standardised and thus, the growth rate for each meningioma will be determined using a joint

For peer review only - http://bmjopen.bmj.com/site/about/guidelines.xhtml

longitudinal and event-time outcomes model which does not require regularly spaced time points, and adjusts for informative follow-up, assuming a different intercept and slope for each meningioma (24, 25). The sum of the regression coefficients of random and fixed effects for the slope estimated from the linear model will best represent the average growth rate for each meningioma. Absolute growth rate (AGR) will be defined as the increase in volume per year in cm^3 whereas relative growth rate (RGR) will be defined as the percentage increase in volume per year.

This statistical analysis plan will be reviewed prior to the final analysis of the study.

Health economic analysis

The health economic analysis will adopt the perspective of the National Health Service in the UK. Costs related to clinic appointments and MRI scans will be calculated for the study cohort's retrospectively performed follow-up plans and compared against two follow-up regimes:

- The follow-up regime proposed by the National Institute for Health Care Excellence (NICE) of 2 scans at 12 months and 5 years
- The follow-up regime using the IMPACT model, stratified by risk of progression

ETHICS AND DISSEMINATION

Study registration

It will be the responsibility of the research team at each unit to register the study as a clinical audit with their hospital's audit department in the UK, including Caldicott guardian or Information Governance approval as required. Overseas sites will register with according to their local institutional policy.

Local investigator responsibilities

The investigator will be responsible for the overall conduct of the study at the site and compliance with the protocol. Responsibilities may be delegated to an appropriate member of

For peer review only - http://bmjopen.bmj.com/site/about/guidelines.xhtml

the local research team. The Investigator must also be familiar with the protocol and the study requirements and it is their responsibility to ensure that all staff assisting with the study are adequately informed about the protocol and the study requirement. The Principal Investigator at each centre is responsible for the quality of the data recorded in the database.

Confidentiality and data protection

No patient identifiable information will be uploaded or stored on the REDCap database. The clinical team can only view the records of patients from their own centre. All records must be identified in a manner designed to maintain patient confidentiality and must be kept in a secure storage area with limited access; all local investigators will store a copy of the link between the patient's unique study number and their patient identifiers on a secure password protected computer, using a blank link file provided by the study team. The investigator and local research terms involved with this study may not disclose or use for any purpose other than performance of the study, any data, record, or other unpublished, confidential information. They also must comply with the requirements of the Data Protection Act 2018 and GDPR with regard to the collection, storage, processing, and disclosure of personal information. Access to collated patient data will be restricted to individuals from the research team and representatives of the sponsor. Computers used to collate the data will have limited access measures via usernames and passwords. Published results will not contain any personal data that could allow identification of individual patients.

Ownership

Ownership of the complete dataset arising from this study resides with the steering committee (named authors in this protocol). Local data collected as part of this study belongs to the local team collecting that data. However, individual clinicians must not submit any part of their individual data for publication or presentation without prior consent from the study research team. Individual participant data, after deidentification, will be made available to researchers whose proposed use of the data is approved by the original study investigators. Proposals should be directed to the primary investigator.

For peer review only - http://bmjopen.bmj.com/site/about/guidelines.xhtml

Dissemination of results

The study results will be reported using the TRIPOD (Transparent Reporting of a multivariable prediction model for Individual Prognosis Or Diagnosis) checklist. The results of this study will be presented at national and international meetings and will be submitted for publication in peer-reviewed journals.

Authorship eligibility

The list of named authors will resemble this protocol's authorship. The contribution of all investigators captured via the REDCap database, will be recognised with PubMed Citable collaborator-status authorship under the umbrella of the IMPACT study investigators.

CONCLUSION

This will be the first international multicentre study collecting data on outcomes of management of incidental asymptomatic intracranial meningioma that will enable external validation of the IMPACT prognostic model and form the basis of ongoing prospective and economic studies.

REFERENCES

1. Cea-Soriano L, Wallander MA, Garcia Rodriguez LA. Epidemiology of meningioma in the United Kingdom. Neuroepidemiology. 2012;39(1):27-34.

2. Ostrom QT, Cioffi G, Gittleman H, Patil N, Waite K, Kruchko C, et al. CBTRUS Statistical Report: Primary Brain and Other Central Nervous System Tumors Diagnosed in the United States in 2012–2016. Neuro Oncol. 2019;21(Supplement_5):v1-v100.

3. Morris Z, Whiteley WN, Longstreth Jr WT, Weber F, Lee YC, Tsushima Y, et al. Incidental findings on brain magnetic resonance imaging: systematic review and meta-analysis. BMJ (Clinical research ed). 2009;339:b3016.

For peer review only - http://bmjopen.bmj.com/site/about/guidelines.xhtml

For peer review only

4. Agarwal V, McCutcheon BA, Hughes JD, Carlson ML, Glasgow AE, Habermann EB, et al. Trends in Management of Intracranial Meningiomas: Analysis of 49,921 Cases from Modern Cohort. World Neurosurg. 2017;106:145-51.

5. Goldbrunner R, Minniti G, Preusser M, Jenkinson MD, Sallabanda K, Houdart E, et al. EANO guidelines for the diagnosis and treatment of meningiomas. The Lancet Oncology. 2016;17(9):e383-91.

6. National Comprehensive Cancer Network. NCCN Clinical Practice Guidelines in Oncology-Central Nervous System Cancer (Version 1.2017) [cited 2017 November 11]. Availabe from: https://www.nccn.org/professionals/physician_gls/pdf/cns.pdf.

7. Islim AI, Mohan M, Moon RDC, Srikandarajah N, Mills SJ, Brodbelt AR, et al. Incidental intracranial meningiomas: a systematic review and meta-analysis of prognostic factors and outcomes. Journal of Neuro-Oncology. 2019;142(2):211-21.

8. Romani R, Ryan G, Benner C, Pollock J. Non-operative meningiomas: long-term follow-up of 136 patients. Acta Neurochir (Wien). 2018.

9. Zeng L, Liang P, Jiao J, Chen J, Lei T. Will an Asymptomatic Meningioma Grow or Not Grow? A Meta-analysis. Journal of neurological surgery Part A, Central European neurosurgery. 2015;76(5):341-7.

10. Behbahani M, Skeie GO, Eide GE, Hausken A, Lund-Johansen M, Skeie BS. A prospective study of the natural history of incidental meningioma—Hold your horses! Neuro-Oncology Practice. 2019;6(6):438-50.

11. Islim AI, Kolamunnage-Dona R, Mohan M, Moon RDC, Crofton A, Haylock BJ, et al. A prognostic model to personalize monitoring regimes for patients with incidental asymptomatic meningiomas. Neuro-Oncology. 2019;22(2):278-89.

12. Kattan MW, Hess KR, Amin MB, Lu Y, Moons KG, Gershenwald JE, et al. American Joint Committee on Cancer acceptance criteria for inclusion of risk models for individualized prognosis in the practice of precision medicine. CA: a cancer journal for clinicians. 2016;66(5):370-4.

13. Vignes JR, Sesay M, Rezajooi K, Gimbert E, Liguoro D. Peritumoral edema and prognosis in intracranial meningioma surgery. J Clin Neurosci. 2008;15(7):764-8.

14. Han MS, Kim YJ, Moon KS, Lee KH, Yang JI, Kang WD, et al. Lessons from surgical outcome for intracranial meningioma involving major venous sinus. Medicine. 2016;95(35).

For peer review only

For peer review only - http://bmjopen.bmj.com/site/about/guidelines.xhtml

15. Cai R, Barnett GH, Novak E, Chao ST, Suh JH. Principal risk of peritumoral edema after stereotactic radiosurgery for intracranial meningioma is tumor-brain contact interface area. Neurosurgery. 2010;66(3):513-22.

16. Kollova A, Liscak R, Novotny J, Jr., Vladyka V, Simonova G, Janouskova L. Gamma Knife surgery for benign meningioma. J Neurosurg. 2007;107(2):325-36.

17. West H, Jin JO. Performance Status in Patients With Cancer. JAMA Oncology. 2015;1(7):998-.

18. Charlson M, Charlson RE, Briggs W, Hollenberg J. Can Disease Management Target Patients Most Likely to Generate High Costs? The Impact of Comorbidity. Journal of General Internal Medicine. 2007;22(4):464-9.

19. Charlson M, Szatrowski TP, Peterson J, Gold J. Validation of a combined comorbidity index. Journal of Clinical Epidemiology. 1994;47(11):1245-51.

20. The Cancer Imaging Archive. Wiki for the VASARI feature set. The National Cancer Institute Web site, https://wiki.cancerimagingarchive.net/display/Public/VASARI+Research+Project. (2015) (Date of access: 06/01/2018).

21. Simpson D. The Recurrence of Intracranial Meningiomas After Surgical Treatment. Journal of Neurology, Neurosurgery & Psychiatry. 1957;20(1):22.

22. Louis DN, Perry A, Reifenberger G, von Deimling A, Figarella-Branger D, Cavenee WK, et al. The 2016 World Health Organization Classification of Tumors of the Central Nervous System: a summary. Acta neuropathologica. 2016;131(6):803-20.

23. Landriel Ibanez FA, Hem S, Ajler P, Vecchi E, Ciraolo C, Baccanelli M, et al. A new classification of complications in neurosurgery. World Neurosurg. 2011;75(5-6):709-15; discussion 604-11.

24. Kolamunnage-Dona R, Williamson PR. Time-dependent efficacy of longitudinal biomarker for clinical endpoint. Statistical Methods in Medical Research. 2016;27(6):1909-24.

25. Williamson PR, Kolamunnage-Dona R, Philipson P, Marson AG. Joint modelling of longitudinal and competing risks data. Statistics in medicine. 2008;27(30):6426-38.

For peer review only - http://bmjopen.bmj.com/site/about/guidelines.xhtml

FOOTNOTES

Authors' contributions

AII, SJM, ARB, MDJ conceived the study. AII drafted the initial study protocol. CPM, RJP, DMF, SM, RK-D, UA, SDK, TG, SJM, ARB, RKM, TS, MDJ provided advice and input on the final protocol. All authors proofread and approved the final manuscript.

Collaborators

IMPACT Study investigators: Kirellos Atef Zaki Abboud, Ahmed Mohamed Abdelfattah, Mohamed Abdelfattah Ateya Abdelhafiz, Abdurrahman Abdelzaher, Eman Abdulwahed, Naeimah Abdussalam, Majd Abouassi, Mahmoud Ibrahim Aboulgheit, Anwaar Abozid, Hudi Abraheem, Suhaib Abualsaud, Mohamed Abuelazm, Amro Abuleil, Randa Wanees Ahmed, Ebtihal Alabed, Ahmed Ababakr Ibrahim Alafandy, Marwa Alaqiliy, Erminia Albanese, Wafa Omar Aldressi, Sarah Wahid Ahmed Abdulghani Alfurais, Heyam Alghazo, Rahaf Alhindi, Tamara Ali, Arousa Ali, Khalil Aliki, Bashair Allawi, Abdulrahman Almjersah, Basil Mahmoud Al-Shami, Salma Alsout, Abdullah Farid Alyousef, Michael Amoo, Mohamed Muftah Anaiba, Omar Saeed Anas, Marwa Anas, Durria Rubat Anwar, Anam Anzak, Sara Rajab Araara, Ahmed Khaled Awad, Mohammed A. Azab, Lamiss Mohamed Abdel Aziz, Ahmed Azzam, Youssef Sameh Badran, Ankur Bajaj, Jarnail Bal, Nadine Ahmed Helmy Mohamed Basha, Marwa Biala, Eva Bourama, Paul Brennan, Daniel Brown, Ferran Brugada-Bellsolà, Placido Bruzzaniti, Annabel Butcher, Mohammad Karam Chaaban, Kevin Ivan Chan, Rebecca Chave-Cox, Yasir Chowdhury, Giles Critchley, Elizabeth Culpin, Bassam Dabbous, Debayan Dasgupta, Katharine J. Drummond, Eman Elbekai, Abdullah Sami Eldaly, Amaal Elgadiam, Abdelazeem Elhabya, Ahmed Sallam Elhawary, Omalez Ahmed Mohmed Elkailani, Omar Elmandouh, Abdelrahman Elsayed, Daisy Evans, Fleur Fisher, Mahmoud El Garhy, Chloé Gelder, Sara Yaser Awni Abu Ghazal, Mohamed Mabrouk Ghonaim, Shamayitri Ghosh, Aimee Goel, Melissa Gough, Fay Greenway, Andrew Gvozdanovic, Abdelrahman Hafez, Alaa Hamdan, Ali Hammed, Hayat Ben Hasan, Najat Shaban Ben Hasan, George Higginbotham, Damian Holliman, Abdelfattah Ibrahim, Omar Abdelnassir Abdelmonem Ismail, Asgeir S. Jakola, Mohsen Javadpour, Sanjeeva Jeyaretna, Josephine Jung, Salah Eddine Oussama Kacimi, Neeraj Kalra, David Kamson, Rahim Abo Kasem, Adham Khalafallah, Sarah Kingdon, Howra Ktayen, Pierfrancesco Lapolla, Jun Yi Lau, Stephen Magill,

For peer review only

Milan Makwana, Hani J. Marcus, Abdulrhman Marwan, Aaron Lawson Mclean, Abhyudaya Mehta, Torstien Meling, Asfand Baig Mirza, Ahmed Attia Mohamed, Mujtaba Mohammad, Korch Moniba, Adel Mouffokes, Ahmed Moussa, Debraj Mukherjee, Ahmed Mahmoud Nafea, Amir H. Zamanipoor Najafabadi, Minh Nguyen, Najwa Noor, Imran Noorani, Olivia Näslund, Nehal Gamal Omar, Abdelrahman Azzam Omran, Setthasorn Zhi Yang Ooi, Mohammed Khalid Osman, Zeinab Othman, Ahmad Ozair, Ana Pardilho, Gildas Patet, Omar Pathmanaban, Andrea Perera, Amit Persad, Jonathan Pollock, Martin Proescholdt, Richard Pullicino, Salma Ramadan, James Robins, Ola Rominiyi, David Rowland, Mahmoud M. Saad, Bobby Sachdev, Fozia Saeed, Abdulrauf Salamah, Ahmed Saleh, Mohamed Saleh, Mahmoud Saleh, Andrea Sangheli, Amira Mohamed Aied Saqr, Ieva Sataite, Esraa Gamal Ahmed Sayed, Verena Schadewaldt, Mohamed Tarek Shaaban, Mohamed Shady, Ahmed Shaheen, Mahmoud Mohamed Mohamed Shalaby, Duaa Shaout, Yara Shatnawi, Mohamed Abdelghany Shehata Shemeis, Zenab Sher, Shoukrie I. Shoukrie, Bente Sandvei Skeie, Alexander Smedley, Agbolahan Sofela, Stuart Stokes, Torbjørn Strømsnes, Hocine Tchouar, Philip Theodosopoulos, Manjul Tripathi, Ismail Ughratdar,Adithya Varma, Anil Varma, Maria Velicu, Sara Venturini, Aminah Werfi, Jacob Young, Catherine Zhang, Taha Zidan, Moaad Zy.

International Consortium on Meningioma (ICOM): Kenneth Aldape, Abdurrahman I. Islim, Karolyn Au, Jill Barnhartz-Sloan, Wenya Linda Bi, Felix Behling, Priscilla K. Brastianos, Chaya Brodie, Nicholas Butowski, Carlos Carlotti, Ana Castro, Aaron Cohen-Gadol, Marta Couce, Michael D. Cusimano, Francesco DiMeco, Katharine J. Drummond, Ian F. Dunn, Craig Erker, Michelle Felicella, Daniel M. Fountain, Evanthia Galanis, Norbert Galldiks, Caterina Giannini, Roland Goldbrunner, Brent Griffith, Rintaro Hashizume, C. Oliver Hanemann, Christel Herold-Mende, Luke Hnenny, Craig Horbinski, Raymond Y. Huang, David James, Michael D. Jenkinson, Christine Jungk, Gerhard Jungwirth, Timothy J. Kaufmann, Boris Krischek, Sylvia Kurz, Daniel Lachance, Christian Lafougère, Katrin Lamszus, Ian Lee, Jeff C. Liu, Serge Makarenko, Tathiana Malta, Yasin Mamatjan, Alireza Mansouri, Christian Mawrin, Michael McDermott, Christopher P. Millward, Jennifer Moliterno-Gunel, Andrew Morokoff, David Munoz, Farshad Nassiri, Houtan Noushmehr, Ho-Keung Ng, Arie Perry, Farhad Pirouzmand, Laila M Poisson, Bianca Pollo, Aditya Ragunathan, David Raleigh, Mirjam Renovanz, Franz Ricklefs, Felix Sahm, Andrea Saladino, Antonio Santacroce, Thomas Santarius, Jens Schittenhelm, Christian Schichor, David Schultz, Nils O. Schmidt, Warren Selman, Helen Shih, Andrew Sloan, Julian Spears, Matija

For peer review only - http://bmjopen.bmj.com/site/about/guidelines.xhtml

Snuderl, James Snyder, Suganth Suppiah, Erik Sulman, Ghazaleh Tabatabai, Marcos Tatagiba, Marco Timmer, Daniela Tirapelli, Joerg C. Tonn, Derek Tsang, Michael A. Vogelbaum, Andreas von Deimling, Tobias Walbert, Simon Walling, Justin Wang, Patrick Y. Wen, Manfred Westphal, Adriana M. Workewych, Stephen Yip, Gabriel Zada, Gelareh Zadeh, Viktor Zherebitskiy.

British Neurosurgical Trainee Research Collaborative (BNTRC): Daniel M. Fountain, Michael T.C. Poon, Angelos Kolias, Julie Woodfield, Rory Piper, Aswin Chari, Abdurrahman I Islim, Neeraj Kalra.

Funding

The authors have not declared a specific grant for this research from any funding agency in the public, commercial or not-for-profit sectors.

Competing interests

AII is supported by Health Education England (North West) Academic Foundation Programme. CPM is a PhD candidate funded by The Brain Tumour Charity to complete The COSMIC project. DMF is funded by a National Institute for Health Research Academic Clinical Fellowship and Cancer Research UK Predoctoral Bursary. TS founded and leads the Anaplastic Meningioma International Consortium (AMiCo). TS and MDJ co-founded the British-Irish Meningioma Society (BIMS). MDJ received a grant from the National Institute for Health Research Health Technology Assessment program for the Radiation versus Observation for Atypical Meningioma (ROAM) trial (NIHR ID: 12/173/14). MDJ and SJM received a grant from the National Institute for Health Research Health Technology Assessment program for Surgeons Trial Of Prophylaxis for Epilepsy in seizure naïve patients with Meningioma (STOP'EM) (NIHR ID: NIHR129748).

Patient and public involvement

Two patient research partners (PRP) have been involved in the design of the study and are members of the steering committee. With the support of the Brain Tumour Charity, represented by a member of the steering committee, the aim is to engage with more PRPs and

For peer review only

to build a partnership to be continued throughout delivery of the study and dissemination and presentation of results.

Patient consent for publication

Not required.

For peer review only

For peer review only - http://bmjopen.bmj.com/site/about/guidelines.xhtml

FIGURES

Figure 1. Process of creating a patient list at each study site

Figure 2. (A-C) T2 MR axial sequences showing the 3 levels of tumour intensity (circle). (A) Hypointense. (B) Isointense. (C) Hyperintense. (D-F) T1-weighted MR with gadolinium (contrast) showing the relationship between the meningioma and the nearby venous sinus (SSS). (D) Separate as there's no clear attachment to the sinus wall. (E) In direct contact with the lateral wall of the sinus. (F) Clear macroscopic distortion and invasion of the sinus.

Figure 3. Study flowchart depicting the process of patient identification and possible management options within the study

For peer review only

For peer review only - http://bmjopen.bmj.com/site/about/guidelines.xhtml

TABLES

Table 1. WHO performance status classification and the age-adjusted Charlson comorbidity index				
WHO performance status classification		Age-adjusted Charlson comorbidity index		
**Score**	**Description**	**Condition**		**weight**
**0**	able to carry out all normal activity without restriction	Age (years)	<50	**0**
**1**	Restricted in strenuous activity but ambulatory and able to carry out light work		50-59	**1**
**2**	Ambulatory and capable of all self-care but unable to carry out any work activities; up and about more than 50% of waking hours		60-69	**2**
**3**	Symptomatic and in a chair or in bed for greater than 50% of the day but not bedridden		70-79	**3**
**4**	Completely disabled; cannot carry out any self-care; totally confined to bed or chair.		≥80	**4**
**5**	Dead	Myocardial infarction		**1**
		Congestive heart failure		**1**
		Peripheral vascular disease		**1**
		Hemiplegia		**2**
		Cerebrovascular disease		**1**
		Pulmonary disease		**1**
		Diabetes		**1**
			With end organ damage	**2**
		Renal disease		**2**
		Liver disease	Mild	**1**
			Severe	**3**
		Peptic ulcer disease		**1**
		Cancer		**2**
			Metastatic	**6**
		Dementia		**1**
		Connective tissue disease		**1**
		AIDS		**6**
		Hypertension		**1**
		Skin ulcers/cellulitis		**2**
		Depression		**1**
		On Warfarin		**1**

For peer review only - http://bmjopen.bmj.com/site/about/guidelines.xhtml

Table 2. Landriel-Ibañez classification of neurosurgical complications	
**Grade I**	Any non–life-threatening deviation from normal postoperative course, not requiring invasive treatment
**Grade Ia**	Complication requiring no drug treatment
**Grade Ib**	Complication requiring drug treatment
**Grade II**	Complication requiring invasive treatment such as surgical, endoscopic, or endovascular interventions
**Grade IIa**	Complication requiring intervention without general anaesthesia
**Grade IIb**	Complication requiring intervention with general anaesthesia
**Grade III**	Life-threatening complications requiring management in ICU
**Grade IIIa**	Complication involving single organ failure
**Grade IIIb**	Complication involving multiple organ failure
**Grade IV**	Complication resulting in death
**Surgical Complications**	Adverse events that are directly related to surgery or surgical technique
**Medical Complications**	Adverse events that are not directly related to surgery or surgical technique
**Suffix "T" (Transient)**	New neurologic deficit improving within 30 days of surgical procedure; can be added to each grade of complication
**Suffix "P" (Persistent)**	New neurologic deficit extending beyond 30 days of surgical procedure; can be added to each grade of complication

For peer review only - http://bmjopen.bmj.com/site/about/guidelines.xhtml

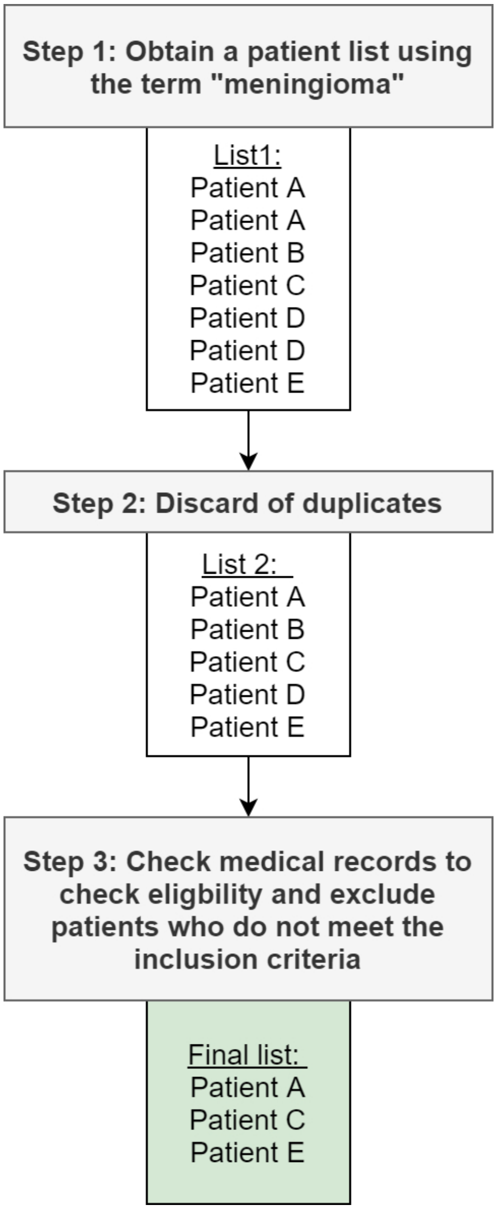

Figure 1. Process of creating a patient list at each study site

170x416mm (72 x 72 DPI)

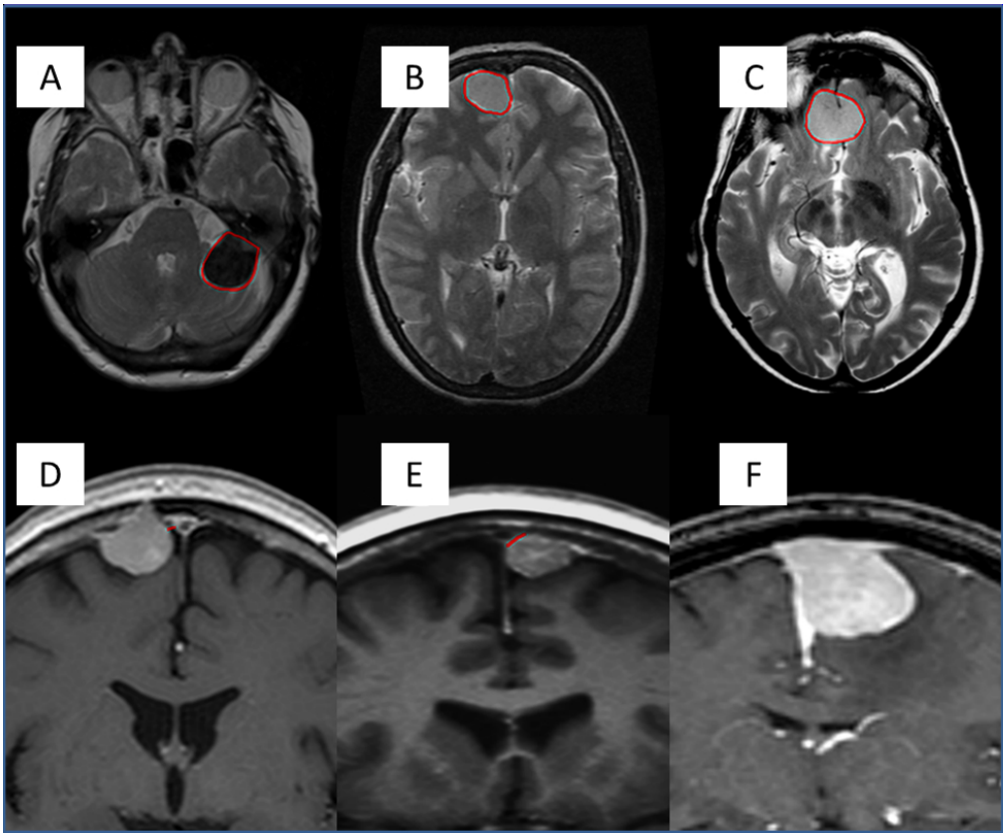

Figure 2. (A-C) T2 MR axial sequences showing the 3 levels of tumour intensity (circle). (A) Hypointense. (B) Isointense. (C) Hyperintense. (D-F) T1-weighted MR with gadolinium (contrast) showing the relationship between the meningioma and the nearby venous sinus (SSS). (D) Separate as there’s no clear attachment to the sinus wall. (E) In direct contact with the lateral wall of the sinus. (F) Clear macroscopic distortion and invasion of the sinus.

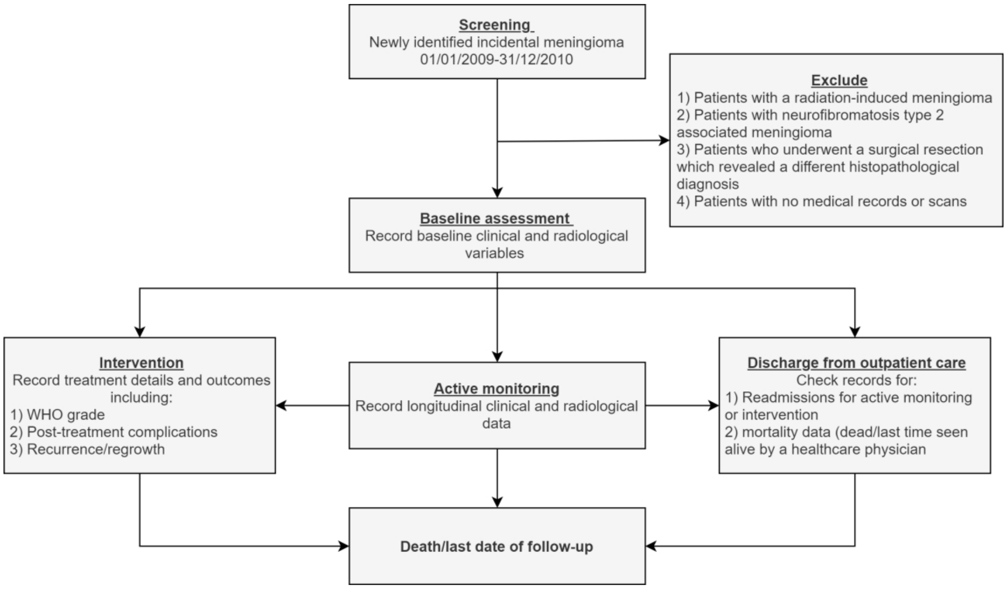

Figure 3. Study flowchart depicting the process of patient identification and possible management options within the study

858x498mm (72 x 72 DPI)

1 APPENDICES

1.1. Appendix. 1. Guide for REDCap database developers

Characteristic	Options	Notes
**BASELINE CLINICAL CHARACTERISTICS (PAGE/SECTION 1)**		
Age (years)	Free field	Required entry
Sex	Dropdown list/check box<br>• Male<br>• Female	
Ethnicity	Dropdown list/check box<br>• White<br>• Mixed / Multiple ethnic groups<br>• Asian / Asian British<br>• Black / African / Caribbean / Black British<br>• Other ethnic group<br>• NA	Required entry. Allow one option only.<br>Other ethic group prompts a free text box
Comorbidities	Check box<br>• Hypertension - systolic > 140 or diastolic > 90 and patients on medical treatment<br>• Previous myocardial infarction<br>• Congestive heart failure<br>• Peripheral vascular disease	Required entry. Allow multiple options.<br>Note to data collector: Age and comorbidities will be used to calculate the age adjusted Charlson comorbidity index. Each of these is defined in the guide provided

	• Previous stroke/TIA - If hemiplegia present, do not check<br>• Hemi/paraplegia<br>• Diabetes which requires medical treatment<br>• Diabetes with end-organ damage - if so, do not check diabetes that requires treatment<br>• COPD/Asthma<br>• Renal disease<br>• Mild liver disease - Hep B/C or cirrhosis without portal hypertension<br>• Moderate to severe liver disease - cirrhosis with portal hypertension, jaundice, ascites<br>• Peptic ulcer disease<br>• Cancer - excluding basal cell carcinoma<br>• Metastatic cancer - if so, do not check cancer<br>• Rheumatic or connective tissue disease<br>• HIV/AIDS<br>• Skin ulcers/cellulitis<br>• Depression<br>• Dementia<br>• On Warfarin	
WHO Performance status	Dropdown list/check box<br><br>• 0<br>• 1<br>• 2	Required entry. Allow one option only

	• 3<br>• 4	
Indication for scan	Dropdown list/check box<br><br>• Headache<br>• Cerebrovascular accident<br>• Head injury<br>• Audiovestibular symptoms<br>• Visual symptoms<br>• Psychiatric symptoms<br>• Cognitive symptoms<br>• Loss of consciousness<br>• Other	Required entry. Allow multiple options.<br><br>Note to data collector: The meningioma must not be thought to be the cause of these symptoms. Other indications might include lethargy, research, sinusitis, anosmia…etc.<br><br>Other prompts a free text box
**NUMBER OF MENINGIOMAS ON 1ST DIAGNOSTIC SCAN (PAGE/SECTION 2)**		
Initial scan date	DD/MM/YYYY	Required entry. YYYY can't be ≤2007 or ≥2011
How many meningiomas?	Check box<br><br>• Single<br>• Multiple	Required entry<br><br>If a single meningioma, direct to section/page 3.<br><br>If multiple, prompt a new entry point (e.g. check box with options being 2-6) with number of meningiomas present. Each meningioma will then be treated as a separate entity with regards to the upcoming sections.
**BASELINE IMAGING CHARACTERISTICS (SECTION/PAGE 3)**		
Meningioma signal intensity on T2	Dropdown list/check box<br><br>• Hypointense<br>• Hyperintense<br>• Isointense<br>• NA	Required entry. Allow one option only<br><br>Note to data collector: In relation to the contralateral grey matter. If only baseline CT available, NA

Meningioma signal intensity on FLAIR	Dropdown list/check box<br><br>• Hypointense<br>• Hyperintense<br>• Isointense<br>• NA	Required entry. Allow one option only<br><br>Note to data collector: In relation to the contralateral grey matter. If only baseline CT available, NA
Peritumoural signal intensity on T2	Dropdown list/check box<br><br>• 0-5%<br>• 6-33%<br>• 34-66%<br>• 67-100%<br>• NA	Required entry. Allow one option only<br><br>Note to data collector: In relation to tumour volume. If only baseline CT available, NA
Peritumoural signal intensity on FLAIR	Dropdown list:<br><br>• 0-5%<br>• 6-33%<br>• 34-66%<br>• 67-100%<br>• NA	Required entry. Allow one option only<br><br>Note to data collector: In relation to tumour volume. If only baseline CT available, NA
Venous sinus nearby	Checkbox	Required entry.<br><br>Note to data collector: Tick box if within 10 mm, in direct contact or invading one of: superior sagittal sinus (SSS), sigmoid sinus (SS), transverse sinus (TS), cavernous sinus (CS) and the confluence of sinuses (CoS)
If yes, specify	Dropdown list/check box<br><br>• Superior sagittal sinus<br>• Cavernous sinus<br>• Sigmoid sinus<br>• Transverse sinus<br>• Confluence of sinuses	Prompt entry if previous option (venous sinus nearby) has been ticked. Allow one option only.
Separate, direct contact or invaded?	Dropdown list/check box<br><br>• Separate	Prompt entry if previous option (venous sinus nearby) has been ticked. Allow one option only

	• Direct contact<br>• Invaded	
In contact with critical neuro-vascular structures?	Checkbox	Required entry
If yes, which	Dropdown list/check box<br><br>• Internal carotid artery<br>• Basilar artery<br>• Vertebral artery<br>• Middle cerebral artery<br>• Anterior cerebral artery<br>• Posterior cerebral artery<br>• Optic apparatus (optic nerve and chiasm)<br>• Trigeminal nerve<br>• Facial nerve<br>• Vestibulo-cochlear nerve<br>• Other	Prompt entry if previous option (In contact with critical neuro-vascular structures?) has been ticked
Major axis (mm)	Free field	Required entry<br><br>Note to data collector: In mm to 1 dp. Measure on axial T1/CT + GAD
Minor axis (mm)	Free field	Required entry<br><br>Note to data collector: In mm to 1 dp. Perpendicular to the major axis on axial T1/CT + GAD
Cor/sag major axis	Free field	Require entry<br><br>Note to data collector: In mm to 1 dp. Maximum height
Location	Dropdown list/Check box<br><br>• Convexity<br>• Parasagittal<br>• Parafalcine	Required entry. Allow one option only<br><br>Note to data collector: as per ICOM classification (appendix 2)

	• Sphenoid wing<br>• Anterior midline<br>• Post fossa-midline<br>• Post fossa-lateral & posterior<br>• Tentorial<br>• Intraventricular<br>• Pineal region	
Location subcategory	Dropdown list/Check box<br><br>• Anterior<br>• Posterior<br>• Falco-tentorial<br>• Lateral<br>• Medial (including ACP)<br>• Cribriform plate/olfactory groove<br>• Planum<br>• Tuberculum/diaphragma sellae<br>• Clival<br>• Petro-clival<br>• Anterior foramen magnum<br>• Petrous<br>• Squamous occipital<br>• Posterior foramen magnum<br>• Supratentorial<br>• Infratentorial	Required entry. Allow one option only<br><br>Note to data collector: ICOM classification (appendix 2)<br><br>Appropriate subcategories will appear based on main category selected
Side	Dropdown list/check box<br><br>• Right<br>• Left<br>• Midline	Required entry. Allow one option only
**MANAGEMENT DECISION (PAGE/SECTION 4)**		

Decision	Dropdown list/Check box<br><br>• Active monitoring<br>• Surgery<br>• SRS<br>• *f*RT<br>• Discharge from outpatient care<br>• Lost to follow-up<br>• Dead	Required entry. Allow one option only<br><br>Note to data collector: You will be directed to the relevant page/section dependent on the choice. If the patient died or was lost to follow-up after this point in time, please choose one of the last two options, albeit the intention might have been to continue monitoring or intervene.
**ACTIVE MONITORING (SECTION/PAGE 5)**		
Scan date	DD/MM/YYYY	Required entry
Peritumoural signal intensity on T2	Dropdown list/check box<br><br>• 0-5%<br>• 6-33%<br>• 34-66%<br>• 67-100%<br>• NA	Required entry. Allow one option only<br><br>Note to data collector: In relation to tumour volume on T2/FLAIR MRI. If only CT or T1 MRI available, NA
Peritumoural signal intensity on FLAIR	Dropdown list/check box<br><br>• 0-5%<br>• 6-33%<br>• 34-66%<br>• 67-100%<br>• NA	Required entry. Allow one option only<br><br>Note to data collector: In relation to tumour volume on FLAIR MRI. If only CT or T1 MRI available, NA
Venous sinus nearby	Checkbox	Required entry.<br><br>Note to data collector: Tick box if within 10 mm, in direct contact or invading one of: superior sagittal sinus (SSS), sigmoid sinus (SS), transverse sinus (TS), cavernous sinus (CS) and the confluence of sinuses (CoS)
If yes, specify	Dropdown list/check box	Prompt entry if previous option (venous sinus nearby) has been ticked. Allow one option only

	• Superior sagittal sinus<br>• Cavernous sinus<br>• Sigmoid sinus<br>• Transverse sinus<br>• Confluence of sinuses	
Separate, direct contact or invaded?	Dropdown list/check box<br><br>• Separate<br>• Direct contact<br>• Invaded	Prompt entry if previous option (venous sinus nearby) has been ticked. Allow one option only
Any new meningioma-related symptoms?	Checkbox	Required entry
If yes, specify domain	Dropdown list/check box<br><br>• Seizure<br>• Headache<br>• Motor<br>• Sensory<br>• Language<br>• Cognitive<br>• Other	Prompt entry if previous option (Any new meningioma-related symptoms?) has been ticked. Allow multiple options
Major axis (mm)	Free field	Required entry<br><br>Note to data collector: In mm to 1 dp. Measure on axial T1/CT + GAD if available
Minor axis (mm)	Free field	Required entry<br><br>Note to data collector: In mm to 1 dp. Perpendicular to the major axis on axial T1/CT + GAD if available
Cor/sag major axis (mm)	Free field	Required entry<br><br>Note to data collector: In mm to 1 dp. Maximum height

Outcome	Dropdown list/check box<br><br>• Resume follow-up (active monitoring)<br>• Surgery<br>• SRS<br>• *f*RT<br>• Discharge<br>• Lost to follow-up<br>• Dead	Required entry. Allow one option only<br><br>Note to data collector: You will be directed to the relevant page/section dependent on the choice. If the patient died or was lost to follow-up after this interval scan, please choose one of the last two options, albeit the intention might have been to continue monitoring or intervene. If the option you choose is set to take place in the future, choose the option, save the data entered and exit the form with no further information required.
**SURGERY (SECTION/PAGE 6)**		
Surgery date	DD/MM/YYYY	Required entry
Indication for intervention	Dropdown list/checkbox<br><br>• Clinical-radiological<br>• Clinical<br>• Radiological<br>• Patient preference	Required entry. Allow one option only
Preoperative WHO PS	Dropdown list/checkbox<br><br>• 0<br>• 1<br>• 2<br>• 3<br>• 4	Required entry. Allow one option only
Preoperative comorbidities	Check box<br><br>• Hypertension - systolic > 140 or diastolic > 90 and patients on medical treatment<br>• Previous myocardial infarction<br>• Congestive heart failure	Required entry. Allow multiple options.<br><br>Note to data collector: Age and comorbidities will be used to calculate the age adjusted Charlson comorbidity index. Each of these is defined in the guide provided

	• Peripheral vascular disease<br>• Previous stroke/TIA - If hemiplegia present, do not check<br>• Hemi/paraplegia<br>• Diabetes which requires medical treatment<br>• Diabetes with end-organ damage - if so, do not check diabetes that requires treatment<br>• COPD/Asthma<br>• Renal disease<br>• Mild liver disease - Hep B/C or cirrhosis without portal hypertension<br>• Moderate to severe liver disease - cirrhosis with portal hypertension, jaundice, ascites<br>• Peptic ulcer disease<br>• Cancer - excluding basal cell carcinoma<br>• Metastatic cancer - if so, do not check cancer<br>• Rheumatic or connective tissue disease<br>• HIV/AIDS<br>• Skin ulcers/cellulitis<br>• Depression<br>• Dementia<br>• On Warfarin	
Simpson grade	Dropdown list/check box<br><br>• 1-GTR<br>• 2-GTR	Required entry. Allow one option only

	• 3-GTR<br>• 4-STR<br>• 5-STR	
WHO grade at the time of surgery	Dropdown list/check box<br><br>• 1<br>• 2<br>• 3	Required entry. Allow one option only<br><br>Note to data collector: According to the WHO classification at the time of surgery
Microscopic brain invasion	Dropdown list/checkbox<br><br>• Yes<br>• No<br>• Brain tissue absent<br>• NA	Required entry. Allow one option only<br><br>Note to data collector: as described in the pathology report.
Updated WHO grade (2016)	Dropdown list/check box<br><br>• 1<br>• 2<br>• 3<br>• NA	Required entry. Allow one option only. Only to appear if date of surgery <2017<br><br>Note to data collector: For meningiomas operated prior to 2016, grading will have been done according to the 2007 classification. The 2016 version can upgrade WHO grade 1 meningiomas to grade 2 if microscopic brain invasion is present. This means that WHO grade 2 and 3 meningiomas remain unchanged. Grade 1 meningiomas on the other hand can be upgraded in the presence of brain invasion. This requires review by a pathologist and so if not feasible, choose NA. For meningiomas classed according to the 2016 WHO classification, grade remains unchanged.
Postoperative surgical complications	Checkbox	Required
Complication	Dropdown list/check box<br><br>• Haemorrhage<br>• Hydrocephalus<br>• Surgical site infection - superficial and deep incisiona<br>• Surgical site infection - intracranial (meningitis, ventriculitis and abscess)	Prompt entry if previous option (Postoperative surgical complications) has been ticked<br><br>Allow more the one option

	• Stroke<br>• CSF leak<br>• Other	
New or worsening neurological impairment	Checkbox	Prompt entry if previous option (Postoperative surgical complications) has been ticked<br><br>Note to data collector: If symptoms present tick box. Note that some patients will have a radiological haemorrhage on postoperative imaging with no symptoms. Include these but don't tick clinical manifestation. On the other hand, some patients will have new symptoms such as seizure with no radiological cause, include these as well.
Clinical manifestation	Dropdown list/checkbox<br><br>• Seizure<br>• Headache<br>• Motor<br>• Sensory<br>• Language<br>• Cognitive<br>• Reduced GCS<br>• Other	Prompt entry if previous option (New or worsening neurological impairment) has been ticked. Allow multiple options
Pharmacological intervention	Checkbox	Prompt entry if previous option (Postoperative surgical complications) has been ticked
Surgical intervention	Dropdown list/checkbox<br><br>• No<br>• Without GA<br>• Under GA	Prompt entry if previous option (Postoperative surgical complications) has been ticked
ICU admission	Checkbox	Prompt entry if previous option (Postoperative surgical complications) has been ticked
Organ failure	Dropdown list/checkbox<br><br>• None<br>• Single-organ<br>• Multi-organ	Prompt entry if previous option (Postoperative surgical complications) has been ticked. Allow one option only

Persisted with no improvement beyond 30 days?	Checkbox	Prompt entry if previous option (Postoperative surgical complications) has been ticked
Postoperative medical complications	checkbox	Required
Complication	Dropdown list/check box<br><br>• Myocardial infarction<br>• Arrhythmia<br>• Pneumonia<br>• Pulmonary embolism<br>• Deep venous thrombosis<br>• Urinary tract infection<br>• Acute kidney injury<br>• Other	Prompt entry if previous option (Postoperative medical complications) has been ticked<br><br>Allow more than one option
Pharmacological intervention	Checkbox	Prompt entry if previous option (Postoperative medical complications) has been ticked
Surgical intervention	Dropdown list/checkbox<br><br>• No<br>• Without GA<br>• Under GA	Prompt entry if previous option (Postoperative medical complications) has been ticked
ICU admission	Checkbox	Prompt entry if previous option (Postoperative medical complications) has been ticked
Organ failure	Dropdown list/checkbox<br><br>• None<br>• Single-organ<br>• Multi-organ	Prompt entry if previous option (Postoperative medical complications) has been ticked. Allow one option only
Persisted with no improvement beyond 30 days?	Checkbox	Prompt entry if previous option (Postoperative medical complications) has been ticked
Postoperative WHO PS	Dropdown list/checkbox<br><br>• 0	Required. Allow one option only

	• 1<br>• 2<br>• 3<br>• 4<br>• 5 (dead)	
Recurrence	Checkbox	Prompt entry if previous option (Postoperative WHO PS) is not 5
Scan date (at recurrence or last follow-up date if no recurrence)	DD/MM/YYYY	Prompt entry if previous option (Postoperative WHO PS) is not 5
WHO PS at time of recurrence/last follow-up	Dropdown list/checkbox<br><br>• 0<br>• 1<br>• 2<br>• 3<br>• 4	Prompt entry if previous option (Postoperative WHO PS) is not 5
**SRS (SECTION/PAGE 7)**		
Pre-radiation WHO PS	Dropdown list/check box<br><br>• 0<br>• 1<br>• 2<br>• 3<br>• 4	Required entry. Allow one option only
Pre-radiation comorbidity	Check box<br><br>• Hypertension - systolic > 140 or diastolic > 90 and patients on medical treatment<br>• Previous myocardial infarction<br>• Congestive heart failure<br>• Peripheral vascular disease	Required entry. Allow multiple options.<br><br>Note to data collector: Age and comorbidities will be used to calculate the age adjusted Charlson comorbidity index. Each of these is defined in the guide provided

	• Previous stroke/TIA - If hemiplegia present, do not check<br>• Hemi/paraplegia<br>• Diabetes which requires medical treatment<br>• Diabetes with end-organ damage - if so, do not check diabetes that requires treatment<br>• COPD/Asthma<br>• Renal disease<br>• Mild liver disease - Hep B/C or cirrhosis without portal hypertension<br>• Moderate to severe liver disease - cirrhosis with portal hypertension, jaundice, ascites<br>• Peptic ulcer disease<br>• Cancer - excluding basal cell carcinoma<br>• Metastatic cancer - if so, do not check cancer<br>• Rheumatic or connective tissue disease<br>• HIV/AIDS<br>• Skin ulcers/cellulitis<br>• Depression<br>• Dementia<br>• On Warfarin	
Dose	Free field	Required entry
Early CTCAE toxicity (≤3 months)	Checkbox	Required entry

Toxicity	Free field	Prompt entry if previous option (Early CTCAE toxicity (≤3 months)) is ticked<br><br>Note to data collector: https://ctep.cancer.gov/protocoldevelopment/electronic_applications/ctc.htm<br><br>Use the following format: System class organ (SOC)-CTCAE term-grade e.g. nervous system disorders-headache-1. Select the complication with the highest toxicity grade
Late CTCAE toxicity	Checkbox	Required entry
Toxicity	Free field	Prompt entry if previous option (Late CTCAE toxicity) is ticked<br><br>Note to data collector: https://ctep.cancer.gov/protocoldevelopment/electronic_applications/ctc.htm<br><br>Use the following format: System class organ (SOC)-CTCAE term-grade e.g. nervous system disorders-headache-1. Select the complication with the highest toxicity grade
Meningioma progression/regrowth	Checkbox	Required
Scan date (at progression or last follow-up date if no progression)	DD/MM/YYYY	Required
WHO PS at time of progression/last follow-up	Dropdown list/checkbox<br><br>• 0<br>• 1<br>• 2<br>• 3<br>• 4<br>• 5 (dead)	Required. Allow one option only
***f*RT (SECTION/PAGE 8)**		

Pre-radiation WHO PS	Dropdown list/check box<br><br>• 0<br>• 1<br>• 2<br>• 3<br>• 4	Required entry
Pre-radiation comorbidity	Check box<br><br>• Myocardial infarction<br>• Congestive heart failure<br>• Peripheral vascular disease<br>• Hemiplegia<br>• Cerebrovascular disease<br>• Pulmonary disease<br>• Diabetes<br>• Renal disease<br>• Liver disease<br>• Peptic ulcer disease<br>• Cancer<br>• Dementia<br>• Connective tissue disease<br>• AIDS<br>• Hypertension<br>• Skin ulcers/cellulitis<br>• Depression<br>• On Warfarin	Required entry. Allow multiple options.<br><br>Note to data collector: Age and comorbidities will be used to calculate the age adjusted Charlson comorbidity index. Each of these is defined in the guide provided
Number of fractions	Free field	Required entry
Fractionated dose	Free field	Required entry
Total dose	Free field	Required entry
Early CTCAE toxicity (≤3 months)	Checkbox	Required entry

Toxicity	Free field	Prompt entry if previous option (Early CTCAE toxicity (≤3 months)) is ticked<br><br>Note to data collector: https://ctep.cancer.gov/protocoldevelopment/electronic_applications/ctc.htm<br><br>Use the following format: System class organ (SOC)-CTCAE term-grade e.g. nervous system disorders-headache-1. Select the complication with the highest toxicity grade
Late CTCAE toxicity	Checkbox	Required entry
Toxicity	Free field	Prompt entry if previous option (Late CTCAE toxicity) is ticked<br><br>Note to data collector: https://ctep.cancer.gov/protocoldevelopment/electronic_applications/ctc.htm<br><br>Use the following format: System class organ (SOC)-CTCAE term-grade e.g. nervous system disorders-headache-1. Select the complication with the highest toxicity grade
Meningioma progression/regrowth	Checkbox	Required
Scan date (at progression or last follow-up date if no progression)	DD/MM/YYYY	Required
WHO PS at time of progression/last follow-up	Dropdown list/checkbox<br><br>• 0<br>• 1<br>• 2<br>• 3<br>• 4<br>• 5 (dead)	Required. Allow one option only
**Discharge from outpatient care/Lost to follow-up (SECTION/PAGE 9)**		

Date of data entry into the database	DD/MM/YYYY	Required entry
Rescanned during the time between discharge/loss to FU and the date of data entry	Checkbox	Required entry
Date of scan	DD/MM/YYYY	Prompt entry if previous option (Readmitted/rescanned during the time between discharge/loss to FU and the date of data entry) has been checked
Reason?	Dropdown list:<br>• Seizure<br>• Headache<br>• Motor<br>• Sensory<br>• Language<br>• Cognitive<br>• Other	Prompt entry if previous option (Readmitted/rescanned during the time between discharge/loss to FU and the date of data entry) has been checked. Allow multiple options
Peritumoural signal intensity on T2	Dropdown list/check box<br>• 0-5%<br>• 6-33%<br>• 34-66%<br>• 67-100%<br>• NA	Prompt entry if previous option (Readmitted/rescanned during the time between discharge/loss to FU and the date of data entry) has been checked. Allow one option only<br>Note to data collector: In relation to tumour volume. If only baseline CT available, NA
Peritumoural signal intensity on FLAIR	Dropdown list:<br>• 0-5%<br>• 6-33%<br>• 34-66%<br>• 67-100%<br>• NA	Prompt entry if previous option (Readmitted/rescanned during the time between discharge/loss to FU and the date of data entry) has been checked. Allow one option only<br>Note to data collector: In relation to tumour volume. If only baseline CT available, NA
Venous sinus nearby	Checkbox<br>• Superior sagittal sinus<br>• Cavernous sinus	Prompt entry if previous option (Readmitted/rescanned during the time between discharge/loss to FU and the date of data entry) has been checked

	• Sigmoid sinus<br>• Transverse sinus<br>• Confluence of sinuses	Note to data collector: Tick box if within 10 mm, in direct contact or invading one of: superior sagittal sinus (SSS), sigmoid sinus (SS), transverse sinus (TS), cavernous sinus (CS) and the confluence of sinuses (CoS)
If yes, specify	Dropdown list/check box	Prompt entry if previous option (venous sinus nearby) has been ticked. Allow one option only
Separate, direct contact or invaded?	Dropdown list/check box<br><br>• Separate<br>• Direct contact<br>• Invaded	Prompt entry if previous option (venous sinus nearby) has been ticked. Allow one option only
Major axis (mm)	Free field	Prompt entry if previous option (Readmitted/rescanned during the time between discharge/loss to FU and the date of data entry) has been checked<br><br>Note to data collector: In mm to 1 dp. Measure on axial T1/CT + GAD
Minor axis (mm)	Free field	Prompt entry if previous option (Readmitted/rescanned during the time between discharge/loss to FU and the date of data entry) has been checked<br><br>Note to data collector: In mm to 1 dp. Perpendicular to the major axis on axial T1/CT + GAD
Cor/sag major axis (mm)	Free field	Prompt entry if previous option (Readmitted/rescanned during the time between discharge/loss to FU and the date of data entry) has been checked<br><br>Note to data collector: In mm to 1 dp. Maximum height
Verdict	Dropdown list/checkbox<br><br>• Related<br>• Unrelated	Prompt entry if previous option (Readmitted/rescanned during the time between discharge/loss to FU and the date of data entry) has been checked. Allow one option only<br><br>Note to data collector: Were the symptoms attributed to the meningioma?
Outcome	Dropdown list/Checkbox	Prompt entry if previous option (Readmitted/rescanned during the time between discharge/loss to FU and the date of data entry) has been checked. Allow one option only

	• Resume follow-up (active monitoring)<br>• Surgery<br>• SRS<br>• *f*RT<br>• Discharge<br>• Lost to follow-up<br>• Dead	Note to data collector: You will be directed to the relevant page/section dependent on the choice. If the patient died or was lost to follow-up after this interval scan, please choose one of the last two options, albeit the intention might have been to continue monitoring or intervene. If the option you choose is set to take place in the future, choose the option, save the data entered and exit the form with no further information required.
Overall outcome	Dropdown list/Checkbox:<br><br>• Dead<br>• Alive	Prompt entry if previous option (Rescanned during the time between discharge/loss to FU and the date of data entry) is not ticked.<br><br>Allow one option only<br><br>Note to data collector: You will be directed to section 10 if Dead is selected.
**Mortality (SECTION/PAGE 10)**		
Date of death	DD/MM/YYYY	Required entry
Cause of death	Dropdown list/checkbox<br><br>• Meningioma-related<br>• Unrelated	Required entry. Allow one option only<br><br>Note to data collector: An example of a meningioma-related death would be for example status epilepticus in a patient who manifested seizures but didn't have treatment. An unrelated death would be for example a community acquired pneumonia. For the purpose of the study, any death occurring from a morbidity, which did not necessitate neurosurgical input/opinion will be classified as unrelated

1.2. Appendix. 2. ICOM classification of meningioma location

ICOM classification system of meningioma locations			
**Main category**	Subcategories		
**Convexity**	Anterior [1]	Posterior[1]	
**Parasagittal**	Anterior[1]	Posterior[1]	Falco-tentorial
**Parafalcine**	Anterior[1]	Posterior[1]	Falco-tentorial
**Sphenoid wing**	Lateral	Medial (including ACP)	
**Anterior midline**	Cribriform plate or olfactory groove[2]	Planum[3]	Tuberculum and diaphragma sellae
**Posterior fossa - midline**	Clival	Petro-clival	Anterior foramen magnum[4]
**Posterior fossa – Lateral & posterior**	Petrous	Squamous occipital	Posterior foramen magnum[4]
**Tentorial**	Supratentorial	Infratentorial	
**Intraventricular**			
**Pineal region[5]**			

[1] The main attachment is located anterior or posterior, respectively, to the coronal suture
[2] Arising between the crista galli and the fronto-sphenoid suture
[3] Arising between the fronto-sphenoid suture and the limbus sphenoidale
[4] The main attachment is located anterior or posterior, respectively, to the hypoglossal canal
[5] No obvious tentorial attachment

For peer review only

BMJ Open

External validation and recalibration of an incidental meningioma prognostic model – IMPACT: protocol for an international multicentre retrospective cohort study

Journal:	*BMJ Open*
Manuscript ID	bmjopen-2021-052705.R1
Article Type:	Protocol
Date Submitted by the Author:	07-Oct-2021

Complete List of Authors:	Islim, Abdurrahman ; University of Liverpool, Institute of Systems, Molecular, & Integrative Biology; The Walton Centre NHS Foundation Trust, Department of Neurosurgery<br>millward, Christopher; University of Liverpool, Institute of Systems, Molecular, & Integrative Biology; The Walton Centre NHS Foundation Trust, Department of Neurosurgery<br>Piper, Rory; John Radcliffe Hospital, Department of Neurosurgery<br>Fountain, Daniel; Salford Royal NHS Foundation Trust, Manchester Centre for Clinical Neurosciences<br>Mehta, Shaveta; Clatterbridge Cancer Centre NHS Foundation Trust, Department of Clinical Oncology; University of Liverpool, Institute of Systems, Molecular, & Integrative Biology<br>Kolamunnage-Dona, Ruwanthi; University of Liverpool, Department of Health Data Science, Institute of Population Health<br>Ali, Usama; University of Oxford, Clinical Trial Service Unit and Epidemiological Studies Unit, Nuffield Department of Population Health<br>Koszdin, Shelli; Veterans Affairs Healthcare System<br>Georgious, Theo; Brain Tumor Charity<br>Mills, Samantha; The Walton Centre NHS Foundation Trust, Department of Neuroradiology<br>Brodbelt, Andrew; University of Liverpool, Institute of Systems, Molecular, & Integrative Biology; The Walton Centre NHS Foundation Trust, Department of Neurosurgery<br>Mathew, Ryan; University of Leeds School of Medicine, Leeds Institute of Medical Research at St James's; Leeds General Infirmary, Department of Neurosurgery<br>Santarius, Thomas; Addenbrooke's Hospital, Department of Neurosurgery; University of Cambridge School of Clinical Medicine, Division of Neurosurgery<br>Jenkinson, Michael; University of Liverpool, Institute of Systems, Molecular, & Integrative Biology; The Walton Centre NHS Foundation Trust, Department of Neurosurgery

**Primary Subject Heading**:	Neurology
Secondary Subject Heading:	Oncology, Evidence based practice
Keywords:	Neurological oncology < ONCOLOGY, NEUROSURGERY, Neuroradiology < NEUROLOGY, ONCOLOGY

SCHOLARONE™
Manuscripts

I, the Submitting Author has the right to grant and does grant on behalf of all authors of the Work (as defined in the below author licence), an exclusive licence and/or a non-exclusive licence for contributions from authors who are: i) UK Crown employees; ii) where BMJ has agreed a CC-BY licence shall apply, and/or iii) in accordance with the terms applicable for US Federal Government officers or employees acting as part of their official duties; on a worldwide, perpetual, irrevocable, royalty-free basis to BMJ Publishing Group Ltd ("BMJ") its licensees and where the relevant Journal is co-owned by BMJ to the co-owners of the Journal, to publish the Work in this journal and any other BMJ products and to exploit all rights, as set out in our licence.

The Submitting Author accepts and understands that any supply made under these terms is made by BMJ to the Submitting Author unless you are acting as an employee on behalf of your employer or a postgraduate student of an affiliated institution which is paying any applicable article publishing charge ("APC") for Open Access articles. Where the Submitting Author wishes to make the Work available on an Open Access basis (and intends to pay the relevant APC), the terms of reuse of such Open Access shall be governed by a Creative Commons licence – details of these licences and which Creative Commons licence will apply to this Work are set out in our licence referred to above.

Other than as permitted in any relevant BMJ Author's Self Archiving Policies, I confirm this Work has not been accepted for publication elsewhere, is not being considered for publication elsewhere and does not duplicate material already published. I confirm all authors consent to publication of this Work and authorise the granting of this licence.

For peer review only

External validation and recalibration of an incidental meningioma prognostic model – IMPACT: protocol for an international multicentre retrospective cohort study

Author and affiliations:

Abdurrahman I. Islim[1,2*], Christopher P. Millward[1,2], Rory J. Piper[3], Daniel M Fountain[4], Shaveta Mehta[1,5], Ruwanthi Kolamunnage-Dona[6], Usama Ali[7], Shelli Diane Koszdin[8], Theo Georgious[9], Samantha J. Mills[10], Andrew R. Brodbelt[1,2], Ryan K. Mathew[11,12], Thomas Santarius[13,14], Michael D. Jenkinson[1,2*] on behalf of the IMPACT Study Investigators, International Consortium on Meningioma (ICOM) and British Neurosurgical Trainee Research Collaborative (BNTRC)

1. Institute of Systems, Molecular, & Integrative Biology, University of Liverpool, Liverpool, UK
2. Department of Neurosurgery, The Walton Centre NHS Foundation Trust, Liverpool, UK
3. Department of Neurosurgery, John Radcliffe Hospital, Oxford University Hospitals NHS Foundation Trust, Oxford, UK
4. Manchester Centre for Clinical Neurosciences, Salford Royal NHS Foundation Trust, Salford, UK
5. Department of Clinical Oncology, The Clatterbridge Cancer Centre NHS Foundation Trust, Wirral, UK
6. Department of Health Data Science, Institute of Population Health, University of Liverpool, Liverpool, UK
7. Clinical Trial Service Unit and Epidemiological Studies Unit, Nuffield Department of Population Health, University of Oxford, Oxford, UK
8. Veterans Affairs Healthcare System, Palo Alto, California, USA
9. The Brain Tumour Charity, Fleet, Hampshire, UK
10. Department of Neuroradiology, The Walton Centre NHS Foundation Trust, Liverpool, UK
11. Leeds Institute of Medical Research at St James's, University of Leeds, Leeds, UK
12. Department of Neurosurgery, Leeds Teaching Hospitals NHS Trust, Leeds, UK
13. Department of Neurosurgery, Addenbrooke's Hospital, Cambridge, UK
14. Division of Neurosurgery, School of Clinical Medicine, University of Cambridge, Cambridge, UK

***Corresponding Authors**

Dr Abdurrahman I Islim, MPhil and Professor Michael D Jenkinson, FRCS, PhD
Institute of Systems, Molecular, & Integrative Biology, University of Liverpool
Department of Neurosurgery, The Walton Centre NHS Foundation Trust
Lower Lane
Liverpool, L9 7LJ
Tel: +44 (0)151 795 5996
Emails: a.islim@doctors.org.uk and Michael.Jenkinson@liverpool.ac.uk

Word count:

Abstract, 355 words; Manuscript, 3613 words; References, 699 words

For peer review only - http://bmjopen.bmj.com/site/about/guidelines.xhtml

ABSTRACT

Introduction: Due to the increased use of computed tomography and magnetic resonance imaging, the prevalence of incidental findings on brain scans is increasing. Meningioma, the commonest primary brain tumour, is a frequently encountered incidental finding, with an estimated prevalence of 3/1000. The management of incidental meningioma varies widely with active clinical-radiological monitoring being the most accepted method by clinicians. Duration of monitoring and time intervals for assessment, however, are not well defined. To this end, we have recently developed a statistical model of progression risk based on single-centre retrospective data. The model – IMPACT (***I***ncidental ***M***eningioma: ***P***rognostic ***A***nalysis Using Patient ***C***omorbidity and MRI ***T***ests) employs baseline clinical and imaging features to categorise the patient with an incidental meningioma into one of three risk groups: low-, medium- and high-risk with a proposed active monitoring strategy based on the risk and temporal trajectory of progression, accounting for actuarial life expectancy. The primary aim of this study is to assess the external validity of this model.

Methods and analysis: IMPACT is a retrospective multi-centre study which will aim to include 1500 patients with an incidental intracranial meningioma, powered to detect a 10% progression risk. Adult patients ≥16 years diagnosed with an incidental meningioma between 01/01/2009 and 31/12/2010 will be included. Clinical and radiological data will be collected longitudinally until the patient reaches one of the study endpoints: intervention (surgery, stereotactic radiosurgery, or fractionated radiotherapy), mortality or last date of follow-up. Data will be uploaded to an online REDCap database with no unique identifiers. External validity of IMPACT will be tested using established statistical methods.

Ethics and dissemination: Local institutional approval at each participating centre will be required. Results of the study will be reported through peer-reviewed articles and conferences and disseminated to participating centres, patients and the public using social media.

Registration details: Researchregistry6051

For peer review only

STRENGTHS AND LIMITATIONS

- The first multi-centre international study to investigate the prognosis of incidental intracranial meningiomas
- The study will include a large cohort of 1500 patients
- The longitudinal study design with serial collection of clinical and imaging data will provide a unique insight into meningioma behaviour and provide a platform for future investigation of novel biomarkers
- The retrospective nature of the study may bias patient and information selection
- Results of the study may be biased by clinician and patient management preference in each participating centre

For peer review only

For peer review only - http://bmjopen.bmj.com/site/about/guidelines.xhtml

INTRODUCTION

Meningiomas have the highest incidence rate amongst all primary central nervous system tumours. Descriptive studies from Europe and North America suggest this rate is between 4.20 and 8.58 per 100,000 individuals (1, 2). Wider access and increased use of magnetic resonance imaging (MRI) and computed tomography (CT) has led to a marked rise in the number of incidental findings in clinical and research settings. Meningiomas comprise 15% of incidental findings on brain MRI and have a prevalence of 3 per 1000 (3). A recent study of the Surveillance, Epidemiology, and End Results (SEER) database demonstrated a substantial increase in the detection of smaller, incidental tumours; between 2004 and 2012, the proportion of meningiomas <1 cm in diameter, diagnosed in a given year, increased in a linear fashion from 6 to 11% (4). Incidental, asymptomatic meningiomas cause patient anxiety and uncertainly around the need for future treatment and often prompt clinicians to commence long-term MRI and clinical follow-up. International consensus guidelines by the European Association of Neuro-Oncology (EANO) and National Comprehensive Cancer Network (NCCN) suggest active monitoring with MRI as first line for managing these tumours (5, 6), but data to advise on the optimal follow-up duration and screening intervals is currently lacking (7).

Previous studies have identified prognostic radiological factors that are associated with the risk of meningioma growth and development of clinical symptoms; yet the timing of such progression is poorly defined (8-10). Moreover, clinical factors such as patient comorbidity and performance status remain unexplored in relation to prognosis but are highly relevant. The patient with an incidental meningioma wants to know whether their tumour will grow and become symptomatic such that it will require safe treatment within their healthy lifetime.

To this end, a recent retrospective cohort study of incidental meningioma patients in the United Kingdom (UK) was conducted to assess the utility of combining routinely available radiological and clinical factors to develop a prognostic model for the risk of incidental meningioma progression during active monitoring (11). The model IMPACT (***I***ncidental ***M***eningioma: ***P***rognostic ***A***nalysis Using Patient ***C***omorbidity and MRI ***T***ests) could be used as a tool to guide active monitoring strategies for patients with an incidental asymptomatic meningioma within the first 10 years of diagnosis, however validation with external datasets is required.

For peer review only - http://bmjopen.bmj.com/site/about/guidelines.xhtml

The primary aim of this international retrospective cohort study of incidental meningioma is to externally validate and calibrate the prognostic model IMPACT, accessible using https://www.impact-meningioma.com. These data will provide insight into the incidence, epidemiology, presentation, management, and long-term outcomes of incidental meningioma, which will inform the development of clinical guidelines and identify areas for future research.

THE IMPACT MODEL

The model, based on MRI parameters, stratifies patients with an incidental meningioma into three risk groups: low-, medium- and high-risk. These MRI parameters are as follows: meningioma volume, meningioma hyperintensity, peritumoral signal change and proximity to critical neurovascular structures. This predictive function was built using an internally validated cox regression model. Patients were also stratified in the model based on age, comorbidity and performance status using competing risk analyses.

OBJECTIVES

Primary objective

To externally validate the prognostic model IMPACT

Secondary objectives

- To update the parameters of the prognostic model IMPACT if measures of external validation demonstrate a poor fit, and internally validate the updated model
- To determine the growth patterns of incidental meningiomas
- To examine the MRI and pathology features of meningiomas subject to surgical resection
- To determine the risk of post-intervention complications and tumour recurrence/growth for meningiomas subject to surgery, stereotactic radiosurgery, or fractionated radiotherapy
- Assess the economic implications of stratifying follow-up according to risk of disease progression

For peer review only - http://bmjopen.bmj.com/site/about/guidelines.xhtml

METHODS AND ANALYSIS

Study design

This will be a retrospective, international multicentre cohort study. The study will include incidental meningioma patients managed at each participating centre. Cases will be identified by the local site research teams using existing patient medical records. Baseline clinical and radiological characteristics, tumour management, and clinical and radiological outcomes will be collected and recorded (anonymised data) on a secure database by the local investigator. Since this study falls within the remit of clinical outcomes audit, individual patient consent is not required. The study will collect data from the medical records for patients newly-diagnosed over a 2-year period between 1st January 2009 and 31st December 2010. This is an observational study and will not alter routine patient care.

Study population and eligibility criteria

The study will include adults (≥16 years of age) with a newly identified incidental intracranial meningioma, as per radiology report, diagnosed between 1st January 2009 and 31st December 2010. Radiological diagnosis is expected to be based on the presence of an extra-axial lesion with broad-based attachment along the dura showing contrast enhancement. The accepted definition of an incidental finding is “a previously undetected abnormality of potential clinical relevance that is unexpectedly discovered and unrelated to the purpose of the examination”.

Exclusion criteria are as follows:

- History of cranial radiation >5 years before diagnosis
- History of neurofibromatosis type 2
- Surgical resection which revealed a different histopathological diagnosis
- Unavailability of medical notes

Patient identification

Eligible patients can be identified using local radiology information systems, for example the Computerised Radiological Information System (CRIS) tool. The search strategy will involve

For peer review only - http://bmjopen.bmj.com/site/about/guidelines.xhtml

review of the medical records of all patients managed with a meningioma at participating centres and exclusion of those that do not meet the selection criteria (Figure 1).

Sample size

For external validation studies, a minimum of 100 events is required (12). The risk of incidental meningioma progression is estimated to be 10% (11). Based on this, data for 1000 patients will be required. To account for variability in the progression risk, follow-up regimes and loss to follow-up, we will aim to include a minimum of 1500 patients across participating centres. An interim analysis will be conducted after data for 500 patients have been collected to assess for the risk of incidental meningioma progression and review the required number of patients.

Study endpoints

Primary endpoint

Disease progression will be defined using a composite endpoint comprising of new symptom development, meningioma-specific mortality, meningioma growth (absolute growth rate ≥2 cm^3/year or absolute growth rate≥1 cm^3/year + relative growth rate ≥30%/year), development or increase of peritumoural brain oedema (defined as increased signal intensity on T2/FLAIR), venous sinus invasion and meningioma volume exceeding 10 cm^3. The first two criteria denote clinical progression while the latter three are related to loss of window of curability. Venous sinus invasion and peritumoural oedema can prevent complete surgical resection (13, 14). Peritumoural oedema and a meningioma volume >10 cm^3 are relative contraindications to stereotactic radiosurgery (15, 16).

Secondary endpoints

Intervention (surgery, stereotactic radiosurgery, or fractionated radiotherapy) and mortality unrelated to the meningioma.

For peer review only - http://bmjopen.bmj.com/site/about/guidelines.xhtml

Data collection

Data will be collected at each centre by members of the local team. Data will be collected from the patient's medical and radiology records. All clinical and radiological information collected for this study by the local investigators should be available routinely and no extra patient assessment will be required. Data will be collected and stored online through a secure University of Liverpool server running the Research Electronic Data Capture (REDCap) web application and using the patient unique study number. Local investigators will be given secure REDCap project server login details. No patient identifiable information will be uploaded or stored on the REDCap database. The study number (site ID_patient ID) is generated by REDCap on creating a new patient record in the database. The clinical team can only view the records of patients from their own centre. All local investigators will store a copy of the link between the patient's unique study number and their patient identifiers on a secure password protected computer, using a blank link file provided by the study team.

REDCap database

REDCap is a secure web application for building and managing online databases. Access to REDCap will be provided by the Liverpool Clinical Trials Centre (LCTC), University of Liverpool, a partner of the REDCap consortium. Database programmers will oversee the development of a data collection tool (Appendix 1) which can be accessed using any electronic device with internet access. The database will be built to comply with the UK's Data Protection Act 2018 and the European Union's General Data Protection Regulation (GDPR). Quality assessment of the tool will be done over two phases. Phase 1 will involve local testing of the tool using pre-existing data (11). Phase 2 will expand testing to three to five additional participating centres. After completion of phase 2, the data collection tool will be made live for use by the participating sites.

For peer review only - http://bmjopen.bmj.com/site/about/guidelines.xhtml

Recorded variables

Baseline clinical variables

Age at diagnosis, sex, ethnicity, the World Health Organisation (WHO) performance status (PS) and the age adjusted Charlson comorbidity index (ACCI) (Table 1) (17-19). These factors will only be recorded at baseline.

Baseline radiological variables

Baseline imaging variables assessed will be:

- Single or multiple intracranial meningioma
- Tumour signal intensity compared to the contralateral grey matter on fluid attenuated inversion recovery (FLAIR) and T2-weighted (T2) MRI (hypo/iso/hyper) (Figure 2)
- Peritumoural signal intensity in relation to tumour volume using the signal change present on FLAIR and T2 MRI (0-5%/6-33%/34-66%/67-100%; adapted from the VASARI [Visually AcceSAble Rembrandt Images] MR features for gliomas (20))
- Meningioma volume using the ABC/2 formula on contrast-enhanced T1-weighted MRI/CT: (A) maximum meningioma diameter on axial plane, (B) diameter perpendicular to (A) and (C) maximum height on coronal/sagittal plane, not taking into the account the dural tail
- Meningioma location classed into non-skull base and skull base and further subcategorised according to the ICOM (International Consortium on Meningioma) classification system (Appendix 2)
- Proximity to major dural venous sinuses (superior sagittal sinus/transverse sinus/sigmoid sinus/cavernous sinus/the confluence of sinuses) categorised as separate (within 10 mm), in direct contact with its wall, or invading, excluding the dural tail (Figure 2)
- Contact with critical neurovascular structures (i.e. internal carotid artery and optic apparatus)

Meningiomas that fulfil one of the two previous categories are said to be in proximity to critical neurovascular structures. A video manual prepared by the study team will be made available to assist with standardisation and quality assurance of scan interpretation across participating centres.

For peer review only - http://bmjopen.bmj.com/site/about/guidelines.xhtml

Management strategy

Management strategies will include active monitoring, intervention (surgery, stereotactic radiosurgery [SRS] and fractionated radiotherapy [*f*RT]) or discharge from outpatient clinic care (Figure 3). **Active monitoring** is defined as regular surveillance imaging and outpatient clinical observation. Recorded factors will include:

- Number of scans, and interval between them (months)
- For each scan: peritumoural signal intensity, venous sinus involvement and meningioma volume
- Each scan will be examined alongside its corresponding outpatient clinic appointment for any evidence of meningioma-related symptoms (motor/sensory/language/cognitive/seizure/headache/other)
- The outcome of each clinical encounter (i.e. outpatient appointment) will be recorded (resume follow-up/surgery/SRS/*f*RT/hospital discharge)

Intervention details; if performed, will also be recorded. These will include indication for intervention (clinical-radiological/clinical/radiological/patient preference) and time to intervention. For patients treated with clinical-radiological or clinical progression, status of meningioma-related neurological morbidity will be noted.

For surgery, the following will additionally be recorded:

- Simpson grade (as recorded by the surgeon in the operative notes) (21)
- WHO grade (classified according to the WHO system in use at the time of surgery and updated according to the WHO 2016 classification dependent on pathologists' availability (22)) and presence of any reported brain invasion (yes / no / not reported)
- Postoperative medical and surgical complications recorded at 30 days (Landriel-Ibañez Classification (Table 2) (23)).
- Follow-up duration (months)
- WHO performance status pre- and postoperatively and at the last follow-up appointment.
- Recurrence on contrast-enhanced MRI during that time (yes/no) and if recurred then the time to recurrence

For SRS and *f*RT:

For peer review only - http://bmjopen.bmj.com/site/about/guidelines.xhtml

- Fractionated dose (*f*RT), number of fractions (*f*RT) and total dose (*f*RT /SRS)
- Early and late (≥3 months) toxicity (assessed by CTCAE v5.0, https://ctep.cancer.gov/protocoldevelopment/electronic_applications/ctc.htm).
- Duration of follow-up post-radiation (months)
- WHO performance status pre- and post-radiation and at the last follow-up appointment
- Progression/regrowth on contrast-enhanced MRI during that time (yes/no) and if progressed/regrew then the time to progression/regrowth

For patients discharged from outpatient care, data sources will be checked for any readmissions/rescans thought to be attributed to the incidental meningioma within the study timeframe; date of diagnosis up to the date of data entry. Outcome following readmissions/rescans will be noted.

Overall outcomes

Overall outcomes by the end of the study period (discharge from outpatient care/lost to follow-up/dead/under on-going active follow-up) and follow-up durations will be recorded. Any deaths encountered during follow-up will be recorded. The medical records for patients who are discharged will also be examined for mortality data.

Data quality assurance

An e-learning module will be prepared by the study team. This will contain the data collection guides and video manual. Upon completion, each study investigator will need to undergo a five-item assessment. An iterative process in which investigators have to redo the assessment or module will dictate their progress as follows:

- An assessment percentage of 100% will indicate successful completion of the module, which will allow the investigator to collect data for the study
- An assessment percentage less 100% will require repeating the assessment
- Five attempts will be allowed
- Subsequent failed attempts will entail review of the module components again

For peer review only - http://bmjopen.bmj.com/site/about/guidelines.xhtml

Planned statistical analysis

Demographic differences across groups will be explored with the χ2 test for categorical variable and the Mann-Whitney U test or Student's t-test for continuous variables. Correlation between baseline variables will be evaluated using the Pearson correlation coefficient. Normally distributed continuous variables will be expressed as mean (standard deviation [SD]) whereas skewed variables as median (interquartile range [IQR]). Differences will be considered statistically significant at $P<0.05$.

External validation

Using IMPACT, the five- and ten-year estimated risk of disease progression for every patient included in this cohort study will be calculated. Kaplan-Meier method will be used to obtain the observed risks. The predictive performance of IMPACT will be assessed by examining measures of calibration and discrimination. Calibration refers to how closely the predicted 5- and 10-year risk of progression agrees with the observed risk. A calibration plot compares the observed and predicted rates of events for each group. A perfect match indicates accurate calibration. The Brier score for censored survival data will also be calculated, which is a measure of accuracy and is the average squared deviation between predicted and observed risk; a lower score represents greater accuracy. Discrimination is the ability of the risk score to differentiate between those patients who do and those who do not experience disease progression during the study timeframe. This measure is quantified by calculating the C-statistic, D-statistic and Chambless and Diao's time-dependent AUC (area under the receiver operating characteristic curve [ROC]) which are tailored towards censored survival data. The proportional hazards assumption of the model will be tested by examination of Schoenfeld residuals, and influential observations will be examined using DFBETA panels.

The two competing risk analyses performed to build the IMPACT model will be repeated with the external dataset and plots of cumulative incidence rate (CIR) and 95% confidence intervals (CIs) will be compared to the original cohort (11). Patients will be split based on WHO PS into two categories: 0-1 and 2-4 and stratified by ACCI (Table 1) into three groups: 0-2, 3-5 and ≥6. The first analysis will assess the CIR of primary intervention at different time points following diagnosis stratified by PS and ACCI groups and the second analysis will evaluate the CIR of disease progression. The competing event for the former will be non-meningioma-specific mortality either observed during follow-up or after being discharged from outpatient care.

For peer review only - http://bmjopen.bmj.com/site/about/guidelines.xhtml

Patients who remain under follow-up will be censored at the last outpatient clinic appointment. Patients discharged alive from outpatient care will censored at the last time they were seen by a healthcare physician up to the date of data entry. For the disease progression analysis, four events will be considered competing in nature, discharge from outpatient care, loss to follow-up, death during follow-up or an intervention before disease progression occurred with the first three grouped together. Censoring will only be done for patients who remain under follow-up at the last clinic appointment. To test the equality across CIR groups, the Fine and Gray test will be carried out.

Model recalibration

If calibration and discrimination measures of external validation demonstrate a poor fit, the model will be recalibrated and adjusted using the data of included patients. This will be done over four stages:

- Stage 1: The regression coefficients will be recalibrated. This will be done using a Cox regression model fitted with the linear predictor as the only covariate
- Stage 2: The recalibrated model predictors will each be removed in a stepwise manner by a non-automated criterion-based procedure starting with the variable with a hazard ratio closest to 1. After removal of this variable, the aforementioned measures of discrimination of calibration and discrimination will be reassessed to detect model improvement. If the performance of the model is unimproved or worsens, the variable will be reintroduced to the model. This step will be repeated in a staged manner until no further improvements are detected. Introduction of new predictive variables will be possible
- Stage 3: The internal validity of the updated model will be assessed using a bootstrapping method
- Stage 4: Adjusted stratification by ACCI and PS (Table 1) will be performed to achieve statistically significant differences in equality across cumulative incidence rate groups, judged by the Fine and Gray test

Additional analyses

We envisage that imaging protocols in the participating centres are varied and non-standardised and thus, the growth rate for each meningioma will be determined using a joint

For peer review only - http://bmjopen.bmj.com/site/about/guidelines.xhtml

longitudinal and event-time outcomes model which does not require regularly spaced time points, and adjusts for informative follow-up, assuming a different intercept and slope for each meningioma (24, 25). The sum of the regression coefficients of random and fixed effects for the slope estimated from the linear model will best represent the average growth rate for each meningioma. Absolute growth rate (AGR) will be defined as the increase in volume per year in cm^3 whereas relative growth rate (RGR) will be defined as the percentage increase in volume per year.

This statistical analysis plan will be reviewed prior to the final analysis of the study.

Health economic analysis

The health economic analysis will adopt the perspective of the National Health Service in the UK. Costs related to clinic appointments and MRI scans will be calculated for the study cohort's retrospectively performed follow-up plans and compared against two follow-up regimes:

- The follow-up regime proposed by the National Institute for Health Care Excellence (NICE) of 2 scans at 12 months and 5 years
- The follow-up regime using the IMPACT model, stratified by risk of progression

Patient and public involvement

Two patient research partners (PRP) have been involved in the design of the study and are members of the steering committee. With the support of the Brain Tumour Charity, represented by a member of the steering committee, the aim is to engage with more PRPs and to build a partnership to be continued throughout delivery of the study and dissemination and presentation of results.

For peer review only

ETHICS AND DISSEMINATION

Study registration

It will be the responsibility of the research team at each unit to register the study as a clinical audit with their hospital's audit department in the UK, including Caldicott guardian or Information Governance approval as required. Overseas sites will register with according to their local institutional policy.

Local investigator responsibilities

The investigator will be responsible for the overall conduct of the study at the site and compliance with the protocol. Responsibilities may be delegated to an appropriate member of the local research team. The Investigator must also be familiar with the protocol and the study requirements and it is their responsibility to ensure that all staff assisting with the study are adequately informed about the protocol and the study requirement. The Principal Investigator at each centre is responsible for the quality of the data recorded in the database.

Confidentiality and data protection

No patient identifiable information will be uploaded or stored on the REDCap database. The clinical team can only view the records of patients from their own centre. All records must be identified in a manner designed to maintain patient confidentiality and must be kept in a secure storage area with limited access; all local investigators will store a copy of the link between the patient's unique study number and their patient identifiers on a secure password protected computer, using a blank link file provided by the study team. The investigator and local research terms involved with this study may not disclose or use for any purpose other than performance of the study, any data, record, or other unpublished, confidential information. They also must comply with the requirements of the Data Protection Act 2018 and GDPR with regard to the collection, storage, processing, and disclosure of personal information. Access to collated patient data will be restricted to individuals from the research team and representatives of the sponsor. Computers used to collate the data will have limited access

For peer review only

measures via usernames and passwords. Published results will not contain any personal data that could allow identification of individual patients.

Ownership

Ownership of the complete dataset arising from this study resides with the steering committee (named authors in this protocol). Local data collected as part of this study belongs to the local team collecting that data. However, individual clinicians must not submit any part of their individual data for publication or presentation without prior consent from the study research team. Individual participant data, after deidentification, will be made available to researchers whose proposed use of the data is approved by the original study investigators. Proposals should be directed to the primary investigator.

Dissemination of results

The study results will be reported using the TRIPOD (Transparent Reporting of a multivariable prediction model for Individual Prognosis Or Diagnosis) checklist. The results of this study will be presented at national and international meetings and will be submitted for publication in peer-reviewed journals.

Authorship eligibility

The list of named authors will resemble this protocol's authorship. The contribution of all investigators captured via the REDCap database, will be recognised with PubMed Citable collaborator-status authorship under the umbrella of the IMPACT study investigators.

REFERENCES

1. Cea-Soriano L, Wallander MA, Garcia Rodriguez LA. Epidemiology of meningioma in the United Kingdom. Neuroepidemiology. 2012;39(1):27-34.

For peer review only - http://bmjopen.bmj.com/site/about/guidelines.xhtml

2. Ostrom QT, Cioffi G, Gittleman H, Patil N, Waite K, Kruchko C, et al. CBTRUS Statistical Report: Primary Brain and Other Central Nervous System Tumors Diagnosed in the United States in 2012–2016. Neuro Oncol. 2019;21(Supplement_5):v1-v100.

3. Morris Z, Whiteley WN, Longstreth Jr WT, Weber F, Lee YC, Tsushima Y, et al. Incidental findings on brain magnetic resonance imaging: systematic review and meta-analysis. BMJ (Clinical research ed). 2009;339:b3016.

4. Agarwal V, McCutcheon BA, Hughes JD, Carlson ML, Glasgow AE, Habermann EB, et al. Trends in Management of Intracranial Meningiomas: Analysis of 49,921 Cases from Modern Cohort. World Neurosurg. 2017;106:145-51.

5. Goldbrunner R, Minniti G, Preusser M, Jenkinson MD, Sallabanda K, Houdart E, et al. EANO guidelines for the diagnosis and treatment of meningiomas. The Lancet Oncology. 2016;17(9):e383-91.

6. National Comprehensive Cancer Network. NCCN Clinical Practice Guidelines in Oncology-Central Nervous System Cancer (Version 1.2017) [cited 2017 November 11]. Availabe from: https://www.nccn.org/professionals/physician_gls/pdf/cns.pdf.

7. Islim AI, Mohan M, Moon RDC, Srikandarajah N, Mills SJ, Brodbelt AR, et al. Incidental intracranial meningiomas: a systematic review and meta-analysis of prognostic factors and outcomes. Journal of Neuro-Oncology. 2019;142(2):211-21.

8. Romani R, Ryan G, Benner C, Pollock J. Non-operative meningiomas: long-term follow-up of 136 patients. Acta Neurochir (Wien). 2018.

9. Zeng L, Liang P, Jiao J, Chen J, Lei T. Will an Asymptomatic Meningioma Grow or Not Grow? A Meta-analysis. Journal of neurological surgery Part A, Central European neurosurgery. 2015;76(5):341-7.

10. Behbahani M, Skeie GO, Eide GE, Hausken A, Lund-Johansen M, Skeie BS. A prospective study of the natural history of incidental meningioma—Hold your horses! Neuro-Oncology Practice. 2019;6(6):438-50.

11. Islim AI, Kolamunnage-Dona R, Mohan M, Moon RDC, Crofton A, Haylock BJ, et al. A prognostic model to personalize monitoring regimes for patients with incidental asymptomatic meningiomas. Neuro-Oncology. 2019;22(2):278-89.

12. Kattan MW, Hess KR, Amin MB, Lu Y, Moons KG, Gershenwald JE, et al. American Joint Committee on Cancer acceptance criteria for inclusion of risk models for individualized

For peer review only

For peer review only - http://bmjopen.bmj.com/site/about/guidelines.xhtml

prognosis in the practice of precision medicine. CA: a cancer journal for clinicians. 2016;66(5):370-4.

13. Vignes JR, Sesay M, Rezajooi K, Gimbert E, Liguoro D. Peritumoral edema and prognosis in intracranial meningioma surgery. J Clin Neurosci. 2008;15(7):764-8.

14. Han MS, Kim YJ, Moon KS, Lee KH, Yang JI, Kang WD, et al. Lessons from surgical outcome for intracranial meningioma involving major venous sinus. Medicine. 2016;95(35).

15. Cai R, Barnett GH, Novak E, Chao ST, Suh JH. Principal risk of peritumoral edema after stereotactic radiosurgery for intracranial meningioma is tumor-brain contact interface area. Neurosurgery. 2010;66(3):513-22.

16. Kollova A, Liscak R, Novotny J, Jr., Vladyka V, Simonova G, Janouskova L. Gamma Knife surgery for benign meningioma. J Neurosurg. 2007;107(2):325-36.

17. West H, Jin JO. Performance Status in Patients With Cancer. JAMA Oncology. 2015;1(7):998-.

18. Charlson M, Charlson RE, Briggs W, Hollenberg J. Can Disease Management Target Patients Most Likely to Generate High Costs? The Impact of Comorbidity. Journal of General Internal Medicine. 2007;22(4):464-9.

19. Charlson M, Szatrowski TP, Peterson J, Gold J. Validation of a combined comorbidity index. Journal of Clinical Epidemiology. 1994;47(11):1245-51.

20. The Cancer Imaging Archive. Wiki for the VASARI feature set. The National Cancer Institute Web site, https://wiki.cancerimagingarchive.net/display/Public/VASARI+Research+Project. (2015) (Date of access: 06/01/2018).

21. Simpson D. The Recurrence of Intracranial Meningiomas After Surgical Treatment. Journal of Neurology, Neurosurgery & Psychiatry. 1957;20(1):22.

22. Louis DN, Perry A, Reifenberger G, von Deimling A, Figarella-Branger D, Cavenee WK, et al. The 2016 World Health Organization Classification of Tumors of the Central Nervous System: a summary. Acta neuropathologica. 2016;131(6):803-20.

23. Landriel Ibanez FA, Hem S, Ajler P, Vecchi E, Ciraolo C, Baccanelli M, et al. A new classification of complications in neurosurgery. World Neurosurg. 2011;75(5-6):709-15; discussion 604-11.

24. Kolamunnage-Dona R, Williamson PR. Time-dependent efficacy of longitudinal biomarker for clinical endpoint. Statistical Methods in Medical Research. 2016;27(6):1909-24.

For peer review only

For peer review only - http://bmjopen.bmj.com/site/about/guidelines.xhtml

25. Williamson PR, Kolamunnage-Dona R, Philipson P, Marson AG. Joint modelling of longitudinal and competing risks data. Statistics in medicine. 2008;27(30):6426-38.

FOOTNOTES

Authors' contributions

AII, SJM, ARB, MDJ conceived the study. AII drafted the initial study protocol. CPM, RJP, DMF, SM, RK-D, UA, SDK, TG, SJM, ARB, RKM, TS, MDJ provided advice and input on the final protocol. All authors proofread and approved the final manuscript.

For peer review only

Collaborators

IMPACT Study investigators: Mohamed Abdelsadg, Suhaib Abualsaud, Amro Abuleil, Kevin Agyemang, Likhith Alakandy, Erminia Albanese, Tamara Ali, Arousa Ali, Anthony Amato-Watkins, Michael Amoo, Mohamed A. R. Arbab, Mutiu Asha, Kareem Austin, Khaled Badran, Jarnail Bal, Chris Barrett, Parameswaran Bhattathiri, Paul Brennan, Jennifer Brown, Daniel Brown, Ferran Brugada-Bellsolà, Placido Bruzzaniti, Annabel Butcher, Rory S. Cairns, Michael Canty, Michael Cearns, Sachiv Chakravarti, Rebecca Chave-Cox, Yasir Chowdhury, Anna Craig-McQuade, Giles Critchley, Peter Crossley, Elizabeth Culpin, Bassam Dabbous, Pedro David Delgado-López, Mohamed Draz, Katharine J. Drummond, Muhammed Elhadi, Ibrahim Elmaadawi, Omar Elmandouh, Daisy Evans, Fleur Fisher, Bryony Ford, Chloé Gelder, Shamayitri Ghosh, Aimee Goel, Melissa Gough, Athanasios Grivas, Andrew Gvozdanovic, Allan Hall, Hytham Hamid, Liv Hartrick, Samih Hassan, Jack Henry, Damian Holliman, Asgeir Jakola, Mohsen Javadpour, Sanjeeva Jeyaretna, Adrian Jimenez, Josephine Jung, Neeraj Kalra, David O. Kamson, Oliver Kennion, Adham M. Khalafallah, Sarah Kingdon, Maya Kommer, Howra Ktayen, Aditaya Kumar, Simon Lammy, Pierfrancesco Lapolla, Jun Yi Lau, Jing Xian Lee, Patricia Littlechild, Darmanin Lora-Kay, Stephen T. Magill, Hani J. Marcus, Fawaz E. Marhoom, Javier Martin-Alonso, Calan Mathieson, Nathan McSorley, Tobias Mederer, Torstien R. Meling, Asfand Baig Mirza, Mujtaba Mohammad, Debraj Mukherjee, Amir H. Zamanipoor Najafabadi, Olivia Näslund, Minh Nguyen, Imran Noorani, Roddy O'Kane, Gildas Patet, Omar N. Pathmanaban, Andrea Perera, Amit Persad, See Yung Phang, Jonathan Pollock, Martin Proescholdt, Richard Pullicino, James Robins, Ola Rominiyi, David Rowland, Bobby Sachdev, Fozia Saeed, Mahmoud Saleh, Ieva Sataite, Verena Schadewaldt, Syed Wajahat Shah, Mustafa El Sheikh, Zenab Sher,

For peer review only - http://bmjopen.bmj.com/site/about/guidelines.xhtml

Bente Sandvei Skeie, Alexander Smedley, Agbolahan Sofela, Jerome St George, Torbjørn Strømsnes, Nigel Suttner, William Taylor, Philip Theodosopoulos, Elliot Tilling, Manjul Tripathi, Ismail Ughratdar, James Ulrich, Anil Varma, Adithya Varma, Maria Velicu, Sara Venturini, Jacob Young, Catherine Zhang.

International Consortium on Meningioma (ICOM): Karolyn Au, Jill Barnholtz-Sloan, Felix Behling, Linda Bi, Priscilla Brastianos, Chaya Brodie, Nicholas Butowski, Ana Castro, Aaron Cohen-Gadol, Marta Couce, Francesco Dimeco, Katherine Drummond, Ian Dunn, Craig Erker, Michelle Felicella, Daniel Fountain, Eva Galanis, Norbert Galldiks, Caterina Giannini, Roland Goldbrunner, Oliver Hanemann, Christel Herold-Mende, Luke Hnenny, Craig Horbinski, Raymond Huang, Abdurrahman I. Islim, Mohsen Javadpour, Michael D. Jenkinson, Christine Jungk, Gerhard Jungwirth, Timothy Kaufmann, Boris Krischek, Sylvia Kurz, Daniel Lachance, Christian Lafougere, Katrin Lamszus, Ian Lee, Serge Makarenko, Tathiana Malta, Tiit Illimar Mathiesen, Christian Mawrin, Michael McDermott, Christopher P. Millward, Jennifer Moliterno-Gunel, Andrew Morokoff, Farshad Nassiri, HK Ng, Houtan Noushmehr, Omar N. Pathmanaban, Arie Perry, Laila Poisson, Bianco Pollo, Aditya Ragunathan, David Raleigh, Mirjam Renovanz, Franz Ricklefs, Felix Sahm, Andrea Saladino, Antonio Santacroce, Thomas Santarius, Christian Schichor, Nils Schimdt, Jens Schittenhelm, Warren Selman, Helen Shih, Andrew Sloan, Matija Snuderl, Jim Snyder, Erik Sulman, Suganth Suppiah, Ghazaleh Tabatabai, Marcos Tatagiba, Marco Timmer, Jorg-Christian Tonn, Michael Vogelbaum, Andreas Von Deimling, Tobias Walbert, Simon Walling, Justin Z. Wang, Patrick Wen, Manfred Westphal, Stephen Yip, Gabriel Zada, Gelareh Zadeh, Viktor Zherebitskiy

British Neurosurgical Trainee Research Collaborative (BNTRC): Daniel M. Fountain, Michael T.C. Poon, Angelos Kolias, Julie Woodfield, Rory Piper, Aswin Chari, Abdurrahman I Islim, Neeraj Kalra.

Funding

The authors have not declared a specific grant for this research from any funding agency in the public, commercial or not-for-profit sectors.

For peer review only

Competing interests

AII is supported by Health Education England (North West) Academic Foundation Programme. CPM is a PhD candidate funded by The Brain Tumour Charity to complete The COSMIC project. DMF is funded by a National Institute for Health Research Academic Clinical Fellowship and Cancer Research UK Predoctoral Bursary. TS founded and leads the Anaplastic Meningioma International Consortium (AMiCo). TS and MDJ co-founded the British-Irish Meningioma Society (BIMS). MDJ received a grant from the National Institute for Health Research Health Technology Assessment program for the Radiation versus Observation for Atypical Meningioma (ROAM) trial (NIHR ID: 12/173/14). MDJ and SJM received a grant from the National Institute for Health Research Health Technology Assessment program for Surgeons Trial Of Prophylaxis for Epilepsy in seizure naïve patients with Meningioma (STOP'EM) (NIHR ID: NIHR129748).

Patient consent for publication

Not required.

For peer review only

FIGURES

Figure 1. Process of creating a patient list at each study site

Figure 2. (A-C) T2 MR axial sequences showing the 3 levels of tumour intensity (circle). (A) Hypointense. (B) Isointense. (C) Hyperintense. (D-F) T1-weighted MR with gadolinium (contrast) showing the relationship between the meningioma and the nearby venous sinus (SSS). (D) Separate as there's no clear attachment to the sinus wall. (E) In direct contact with the lateral wall of the sinus. (F) Clear macroscopic distortion and invasion of the sinus.

Figure 3. Study flowchart depicting the process of patient identification and possible management options within the study

For peer review only

For peer review only - http://bmjopen.bmj.com/site/about/guidelines.xhtml

TABLES

Table 1. WHO performance status classification and the age-adjusted Charlson comorbidity index

WHO performance status classification		Age-adjusted Charlson comorbidity index		
**Score**	**Description**	**Condition**		**weight**
**0**	able to carry out all normal activity without restriction	Age (years)	<50	**0**
**1**	Restricted in strenuous activity but ambulatory and able to carry out light work		50-59	**1**
**2**	Ambulatory and capable of all self-care but unable to carry out any work activities; up and about more than 50% of waking hours		60-69	**2**
**3**	Symptomatic and in a chair or in bed for greater than 50% of the day but not bedridden		70-79	**3**
**4**	Completely disabled; cannot carry out any self-care; totally confined to bed or chair.		≥80	**4**
**5**	Dead	Myocardial infarction		**1**
		Congestive heart failure		**1**
		Peripheral vascular disease		**1**
		Hemiplegia		**2**
		Cerebrovascular disease		**1**
		Pulmonary disease		**1**
		Diabetes		**1**
			With end organ damage	**2**
		Renal disease		**2**
		Liver disease	Mild	**1**
			Severe	**3**
		Peptic ulcer disease		**1**
		Cancer		**2**
			Metastatic	**6**
		Dementia		**1**
		Connective tissue disease		**1**
		AIDS		**6**
		Hypertension		**1**
		Skin ulcers/cellulitis		**2**
		Depression		**1**
		On Warfarin		**1**

For peer review only

For peer review only - http://bmjopen.bmj.com/site/about/guidelines.xhtml

Table 2. Landriel-Ibañez classification of neurosurgical complications	
**Grade I**	Any non–life-threatening deviation from normal postoperative course, not requiring invasive treatment
**Grade Ia**	Complication requiring no drug treatment
**Grade Ib**	Complication requiring drug treatment
**Grade II**	Complication requiring invasive treatment such as surgical, endoscopic, or endovascular interventions
**Grade IIa**	Complication requiring intervention without general anaesthesia
**Grade IIb**	Complication requiring intervention with general anaesthesia
**Grade III**	Life-threatening complications requiring management in ICU
**Grade IIIa**	Complication involving single organ failure
**Grade IIIb**	Complication involving multiple organ failure
**Grade IV**	Complication resulting in death
**Surgical Complications**	Adverse events that are directly related to surgery or surgical technique
**Medical Complications**	Adverse events that are not directly related to surgery or surgical technique
**Suffix "T" (Transient)**	New neurologic deficit improving within 30 days of surgical procedure; can be added to each grade of complication
**Suffix "P" (Persistent)**	New neurologic deficit extending beyond 30 days of surgical procedure; can be added to each grade of complication

For peer review only - http://bmjopen.bmj.com/site/about/guidelines.xhtml

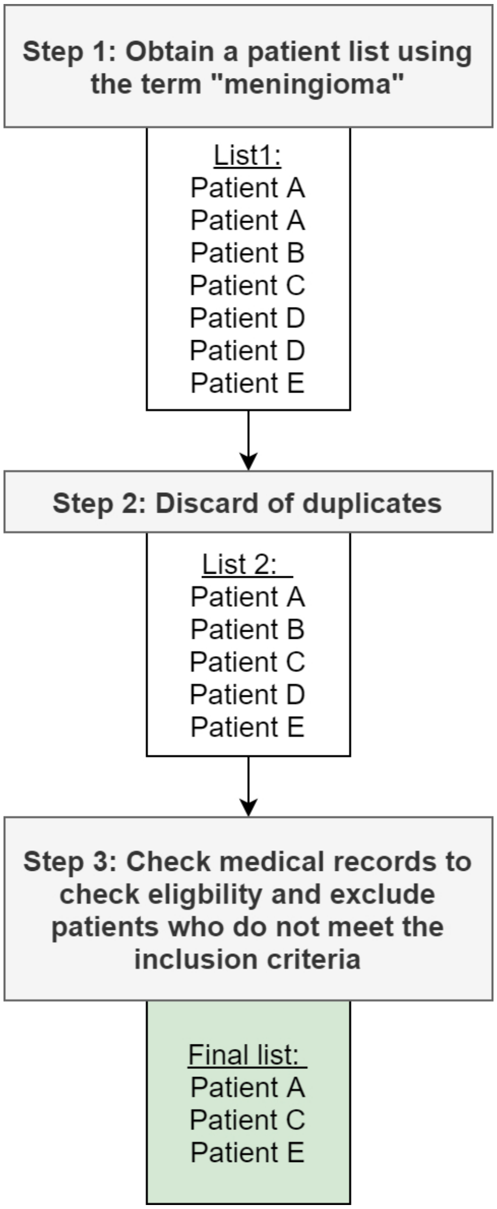

Figure 1. Process of creating a patient list at each study site

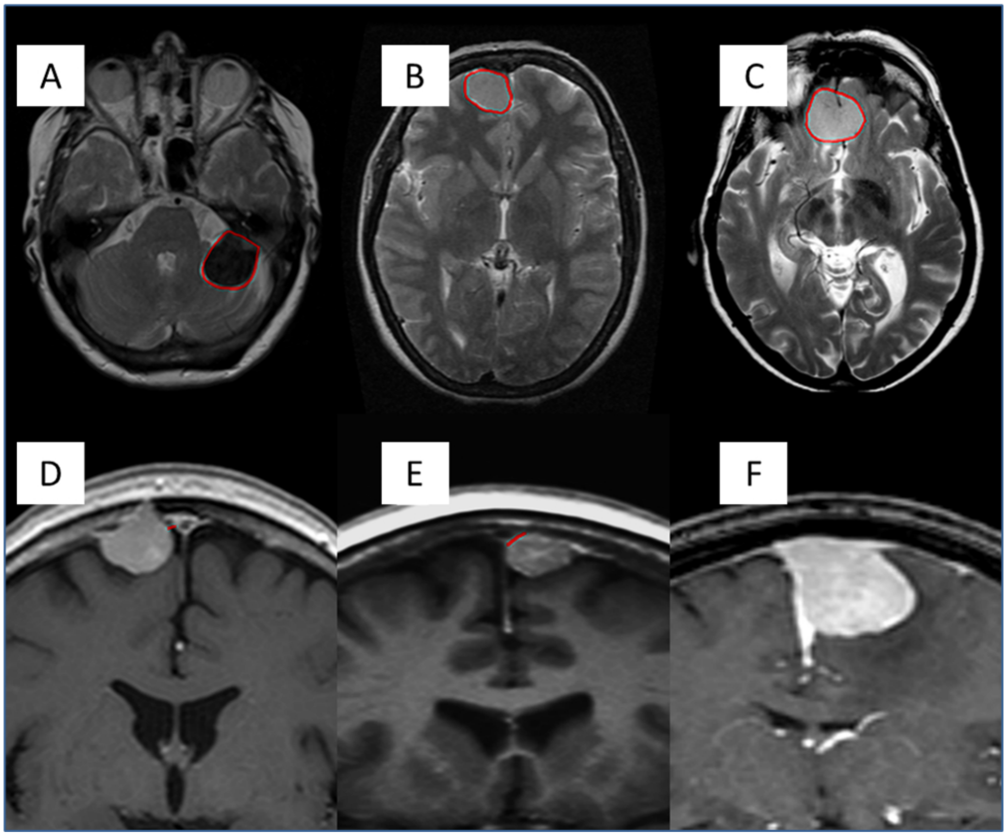

Figure 2. (A-C) T2 MR axial sequences showing the 3 levels of tumour intensity (circle). (A) Hypointense. (B) Isointense. (C) Hyperintense. (D-F) T1-weighted MR with gadolinium (contrast) showing the relationship between the meningioma and the nearby venous sinus (SSS). (D) Separate as there's no clear attachment to the sinus wall. (E) In direct contact with the lateral wall of the sinus. (F) Clear macroscopic distortion and invasion of the sinus.

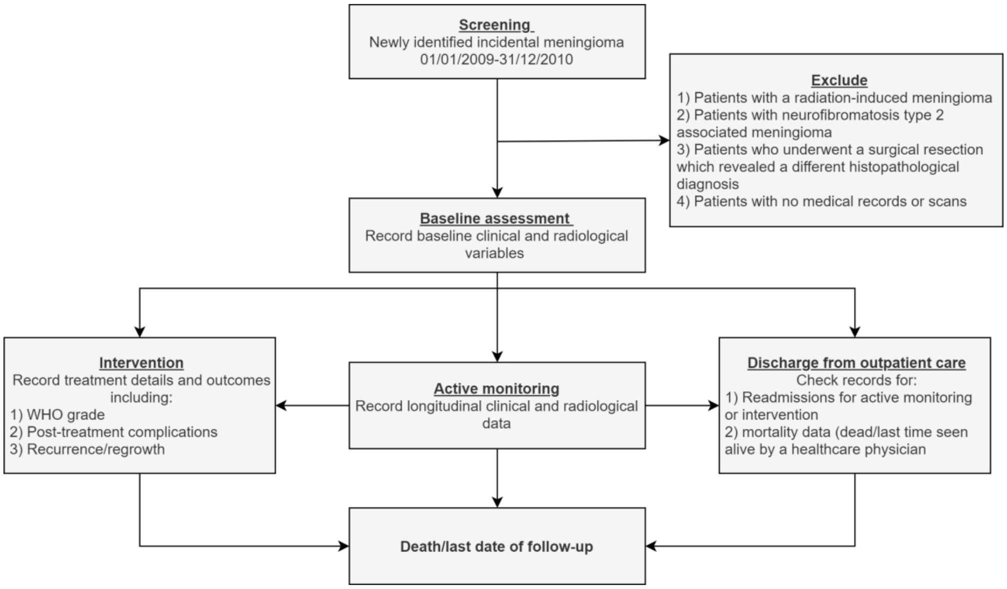

Figure 3. Study flowchart depicting the process of patient identification and possible management options within the study

1 APPENDICES

1.1. Appendix. 1. Guide for REDCap database developers

Characteristic	Options	Notes
**BASELINE CLINICAL CHARACTERISTICS (PAGE/SECTION 1)**		
Age (years)	Free field	Required entry
Sex	Dropdown list/check box<br>• Male<br>• Female	
Ethnicity	Dropdown list/check box<br>• White<br>• Mixed / Multiple ethnic groups<br>• Asian / Asian British<br>• Black / African / Caribbean / Black British<br>• Other ethnic group<br>• NA	Required entry. Allow one option only.<br>Other ethic group prompts a free text box
Comorbidities	Check box<br>• Hypertension - systolic > 140 or diastolic > 90 and patients on medical treatment<br>• Previous myocardial infarction<br>• Congestive heart failure<br>• Peripheral vascular disease	Required entry. Allow multiple options.<br>Note to data collector: Age and comorbidities will be used to calculate the age adjusted Charlson comorbidity index. Each of these is defined in the guide provided

	• Previous stroke/TIA - If hemiplegia present, do not check<br>• Hemi/paraplegia<br>• Diabetes which requires medical treatment<br>• Diabetes with end-organ damage - if so, do not check diabetes that requires treatment<br>• COPD/Asthma<br>• Renal disease<br>• Mild liver disease - Hep B/C or cirrhosis without portal hypertension<br>• Moderate to severe liver disease - cirrhosis with portal hypertension, jaundice, ascites<br>• Peptic ulcer disease<br>• Cancer - excluding basal cell carcinoma<br>• Metastatic cancer - if so, do not check cancer<br>• Rheumatic or connective tissue disease<br>• HIV/AIDS<br>• Skin ulcers/cellulitis<br>• Depression<br>• Dementia<br>• On Warfarin	
WHO Performance status	Dropdown list/check box<br><br>• 0<br>• 1<br>• 2	Required entry. Allow one option only

	• 3<br>• 4	
Indication for scan	Dropdown list/check box<br><br>• Headache<br>• Cerebrovascular accident<br>• Head injury<br>• Audiovestibular symptoms<br>• Visual symptoms<br>• Psychiatric symptoms<br>• Cognitive symptoms<br>• Loss of consciousness<br>• Other	Required entry. Allow multiple options.<br><br>Note to data collector: The meningioma must not be thought to be the cause of these symptoms. Other indications might include lethargy, research, sinusitis, anosmia…etc.<br><br>Other prompts a free text box
**NUMBER OF MENINGIOMAS ON 1ST DIAGNOSTIC SCAN (PAGE/SECTION 2)**		
Initial scan date	DD/MM/YYYY	Required entry. YYYY can't be ≤2007 or ≥2011
How many meningiomas?	Check box<br><br>• Single<br>• Multiple	Required entry<br><br>If a single meningioma, direct to section/page 3.<br><br>If multiple, prompt a new entry point (e.g. check box with options being 2-6) with number of meningiomas present. Each meningioma will then be treated as a separate entity with regards to the upcoming sections.
**BASELINE IMAGING CHARACTERISTICS (SECTION/PAGE 3)**		
Meningioma signal intensity on T2	Dropdown list/check box<br><br>• Hypointense<br>• Hyperintense<br>• Isointense<br>• NA	Required entry. Allow one option only<br><br>Note to data collector: In relation to the contralateral grey matter. If only baseline CT available, NA

Meningioma signal intensity on FLAIR	Dropdown list/check box<br><br>• Hypointense<br>• Hyperintense<br>• Isointense<br>• NA	Required entry. Allow one option only<br><br>Note to data collector: In relation to the contralateral grey matter. If only baseline CT available, NA
Peritumoural signal intensity on T2	Dropdown list/check box<br><br>• 0-5%<br>• 6-33%<br>• 34-66%<br>• 67-100%<br>• NA	Required entry. Allow one option only<br><br>Note to data collector: In relation to tumour volume. If only baseline CT available, NA
Peritumoural signal intensity on FLAIR	Dropdown list:<br><br>• 0-5%<br>• 6-33%<br>• 34-66%<br>• 67-100%<br>• NA	Required entry. Allow one option only<br><br>Note to data collector: In relation to tumour volume. If only baseline CT available, NA
Venous sinus nearby	Checkbox	Required entry.<br><br>Note to data collector: Tick box if within 10 mm, in direct contact or invading one of: superior sagittal sinus (SSS), sigmoid sinus (SS), transverse sinus (TS), cavernous sinus (CS) and the confluence of sinuses (CoS)
If yes, specify	Dropdown list/check box<br><br>• Superior sagittal sinus<br>• Cavernous sinus<br>• Sigmoid sinus<br>• Transverse sinus<br>• Confluence of sinuses	Prompt entry if previous option (venous sinus nearby) has been ticked. Allow one option only.
Separate, direct contact or invaded?	Dropdown list/check box<br><br>• Separate	Prompt entry if previous option (venous sinus nearby) has been ticked. Allow one option only

	• Direct contact<br>• Invaded	
In contact with critical neuro-vascular structures?	Checkbox	Required entry
If yes, which	Dropdown list/check box<br><br>• Internal carotid artery<br>• Basilar artery<br>• Vertebral artery<br>• Middle cerebral artery<br>• Anterior cerebral artery<br>• Posterior cerebral artery<br>• Optic apparatus (optic nerve and chiasm)<br>• Trigeminal nerve<br>• Facial nerve<br>• Vestibulo-cochlear nerve<br>• Other	Prompt entry if previous option (In contact with critical neuro-vascular structures?) has been ticked
Major axis (mm)	Free field	Required entry<br><br>Note to data collector: In mm to 1 dp. Measure on axial T1/CT + GAD
Minor axis (mm)	Free field	Required entry<br><br>Note to data collector: In mm to 1 dp. Perpendicular to the major axis on axial T1/CT + GAD
Cor/sag major axis	Free field	Require entry<br><br>Note to data collector: In mm to 1 dp. Maximum height
Location	Dropdown list/Check box<br><br>• Convexity<br>• Parasagittal<br>• Parafalcine	Required entry. Allow one option only<br><br>Note to data collector: as per ICOM classification (appendix 2)

	• Sphenoid wing<br>• Anterior midline<br>• Post fossa-midline<br>• Post fossa-lateral & posterior<br>• Tentorial<br>• Intraventricular<br>• Pineal region	
Location subcategory	Dropdown list/Check box<br><br>• Anterior<br>• Posterior<br>• Falco-tentorial<br>• Lateral<br>• Medial (including ACP)<br>• Cribriform plate/olfactory groove<br>• Planum<br>• Tuberculum/diaphragma sellae<br>• Clival<br>• Petro-clival<br>• Anterior foramen magnum<br>• Petrous<br>• Squamous occipital<br>• Posterior foramen magnum<br>• Supratentorial<br>• Infratentorial	Required entry. Allow one option only<br><br>Note to data collector: ICOM classification (appendix 2)<br><br>Appropriate subcategories will appear based on main category selected
Side	Dropdown list/check box<br><br>• Right<br>• Left<br>• Midline	Required entry. Allow one option only
**MANAGEMENT DECISION (PAGE/SECTION 4)**		

Decision	Dropdown list/Check box<br><br>• Active monitoring<br>• Surgery<br>• SRS<br>• *f*RT<br>• Discharge from outpatient care<br>• Lost to follow-up<br>• Dead	Required entry. Allow one option only<br><br>Note to data collector: You will be directed to the relevant page/section dependent on the choice. If the patient died or was lost to follow-up after this point in time, please choose one of the last two options, albeit the intention might have been to continue monitoring or intervene.
**ACTIVE MONITORING (SECTION/PAGE 5)**		
Scan date	DD/MM/YYYY	Required entry
Peritumoural signal intensity on T2	Dropdown list/check box<br><br>• 0-5%<br>• 6-33%<br>• 34-66%<br>• 67-100%<br>• NA	Required entry. Allow one option only<br><br>Note to data collector: In relation to tumour volume on T2/FLAIR MRI. If only CT or T1 MRI available, NA
Peritumoural signal intensity on FLAIR	Dropdown list/check box<br><br>• 0-5%<br>• 6-33%<br>• 34-66%<br>• 67-100%<br>• NA	Required entry. Allow one option only<br><br>Note to data collector: In relation to tumour volume on FLAIR MRI. If only CT or T1 MRI available, NA
Venous sinus nearby	Checkbox	Required entry.<br><br>Note to data collector: Tick box if within 10 mm, in direct contact or invading one of: superior sagittal sinus (SSS), sigmoid sinus (SS), transverse sinus (TS), cavernous sinus (CS) and the confluence of sinuses (CoS)
If yes, specify	Dropdown list/check box	Prompt entry if previous option (venous sinus nearby) has been ticked. Allow one option only

	• Superior sagittal sinus<br>• Cavernous sinus<br>• Sigmoid sinus<br>• Transverse sinus<br>• Confluence of sinuses	
Separate, direct contact or invaded?	Dropdown list/check box<br><br>• Separate<br>• Direct contact<br>• Invaded	Prompt entry if previous option (venous sinus nearby) has been ticked. Allow one option only
Any new meningioma-related symptoms?	Checkbox	Required entry
If yes, specify domain	Dropdown list/check box<br><br>• Seizure<br>• Headache<br>• Motor<br>• Sensory<br>• Language<br>• Cognitive<br>• Other	Prompt entry if previous option (Any new meningioma-related symptoms?) has been ticked. Allow multiple options
Major axis (mm)	Free field	Required entry<br><br>Note to data collector: In mm to 1 dp. Measure on axial T1/CT + GAD if available
Minor axis (mm)	Free field	Required entry<br><br>Note to data collector: In mm to 1 dp. Perpendicular to the major axis on axial T1/CT + GAD if available
Cor/sag major axis (mm)	Free field	Required entry<br><br>Note to data collector: In mm to 1 dp. Maximum height

Outcome	Dropdown list/check box<br><br>• Resume follow-up (active monitoring)<br>• Surgery<br>• SRS<br>• *f*RT<br>• Discharge<br>• Lost to follow-up<br>• Dead	Required entry. Allow one option only<br><br>Note to data collector: You will be directed to the relevant page/section dependent on the choice. If the patient died or was lost to follow-up after this interval scan, please choose one of the last two options, albeit the intention might have been to continue monitoring or intervene. If the option you choose is set to take place in the future, choose the option, save the data entered and exit the form with no further information required.
**SURGERY (SECTION/PAGE 6)**		
Surgery date	DD/MM/YYYY	Required entry
Indication for intervention	Dropdown list/checkbox<br><br>• Clinical-radiological<br>• Clinical<br>• Radiological<br>• Patient preference	Required entry. Allow one option only
Preoperative WHO PS	Dropdown list/checkbox<br><br>• 0<br>• 1<br>• 2<br>• 3<br>• 4	Required entry. Allow one option only
Preoperative comorbidities	Check box<br><br>• Hypertension - systolic > 140 or diastolic > 90 and patients on medical treatment<br>• Previous myocardial infarction<br>• Congestive heart failure	Required entry. Allow multiple options.<br><br>Note to data collector: Age and comorbidities will be used to calculate the age adjusted Charlson comorbidity index. Each of these is defined in the guide provided

	• Peripheral vascular disease<br>• Previous stroke/TIA - If hemiplegia present, do not check<br>• Hemi/paraplegia<br>• Diabetes which requires medical treatment<br>• Diabetes with end-organ damage - if so, do not check diabetes that requires treatment<br>• COPD/Asthma<br>• Renal disease<br>• Mild liver disease - Hep B/C or cirrhosis without portal hypertension<br>• Moderate to severe liver disease - cirrhosis with portal hypertension, jaundice, ascites<br>• Peptic ulcer disease<br>• Cancer - excluding basal cell carcinoma<br>• Metastatic cancer - if so, do not check cancer<br>• Rheumatic or connective tissue disease<br>• HIV/AIDS<br>• Skin ulcers/cellulitis<br>• Depression<br>• Dementia<br>• On Warfarin	
Simpson grade	Dropdown list/check box<br><br>• 1-GTR<br>• 2-GTR	Required entry. Allow one option only

	• 3-GTR<br>• 4-STR<br>• 5-STR	
WHO grade at the time of surgery	Dropdown list/check box<br><br>• 1<br>• 2<br>• 3	Required entry. Allow one option only<br><br>Note to data collector: According to the WHO classification at the time of surgery
Microscopic brain invasion	Dropdown list/checkbox<br><br>• Yes<br>• No<br>• Brain tissue absent<br>• NA	Required entry. Allow one option only<br><br>Note to data collector: as described in the pathology report.
Updated WHO grade (2016)	Dropdown list/check box<br><br>• 1<br>• 2<br>• 3<br>• NA	Required entry. Allow one option only. Only to appear if date of surgery <2017<br><br>Note to data collector: For meningiomas operated prior to 2016, grading will have been done according to the 2007 classification. The 2016 version can upgrade WHO grade 1 meningiomas to grade 2 if microscopic brain invasion is present. This means that WHO grade 2 and 3 meningiomas remain unchanged. Grade 1 meningiomas on the other hand can be upgraded in the presence of brain invasion. This requires review by a pathologist and so if not feasible, choose NA. For meningiomas classed according to the 2016 WHO classification, grade remains unchanged.
Postoperative surgical complications	Checkbox	Required
Complication	Dropdown list/check box<br><br>• Haemorrhage<br>• Hydrocephalus<br>• Surgical site infection - superficial and deep incisiona<br>• Surgical site infection - intracranial (meningitis, ventriculitis and abscess)	Prompt entry if previous option (Postoperative surgical complications) has been ticked<br><br>Allow more the one option

	• Stroke<br>• CSF leak<br>• Other	
New or worsening neurological impairment	Checkbox	Prompt entry if previous option (Postoperative surgical complications) has been ticked<br><br>Note to data collector: If symptoms present tick box. Note that some patients will have a radiological haemorrhage on postoperative imaging with no symptoms. Include these but don't tick clinical manifestation. On the other hand, some patients will have new symptoms such as seizure with no radiological cause, include these as well.
Clinical manifestation	Dropdown list/checkbox<br><br>• Seizure<br>• Headache<br>• Motor<br>• Sensory<br>• Language<br>• Cognitive<br>• Reduced GCS<br>• Other	Prompt entry if previous option (New or worsening neurological impairment) has been ticked. Allow multiple options
Pharmacological intervention	Checkbox	Prompt entry if previous option (Postoperative surgical complications) has been ticked
Surgical intervention	Dropdown list/checkbox<br><br>• No<br>• Without GA<br>• Under GA	Prompt entry if previous option (Postoperative surgical complications) has been ticked
ICU admission	Checkbox	Prompt entry if previous option (Postoperative surgical complications) has been ticked
Organ failure	Dropdown list/checkbox<br><br>• None<br>• Single-organ<br>• Multi-organ	Prompt entry if previous option (Postoperative surgical complications) has been ticked. Allow one option only

Persisted with no improvement beyond 30 days?	Checkbox	Prompt entry if previous option (Postoperative surgical complications) has been ticked
Postoperative medical complications	checkbox	Required
Complication	Dropdown list/check box<br><br>• Myocardial infarction<br>• Arrhythmia<br>• Pneumonia<br>• Pulmonary embolism<br>• Deep venous thrombosis<br>• Urinary tract infection<br>• Acute kidney injury<br>• Other	Prompt entry if previous option (Postoperative medical complications) has been ticked<br><br>Allow more than one option
Pharmacological intervention	Checkbox	Prompt entry if previous option (Postoperative medical complications) has been ticked
Surgical intervention	Dropdown list/checkbox<br><br>• No<br>• Without GA<br>• Under GA	Prompt entry if previous option (Postoperative medical complications) has been ticked
ICU admission	Checkbox	Prompt entry if previous option (Postoperative medical complications) has been ticked
Organ failure	Dropdown list/checkbox<br><br>• None<br>• Single-organ<br>• Multi-organ	Prompt entry if previous option (Postoperative medical complications) has been ticked. Allow one option only
Persisted with no improvement beyond 30 days?	Checkbox	Prompt entry if previous option (Postoperative medical complications) has been ticked
Postoperative WHO PS	Dropdown list/checkbox<br><br>• 0	Required. Allow one option only

	• 1<br>• 2<br>• 3<br>• 4<br>• 5 (dead)	
Recurrence	Checkbox	Prompt entry if previous option (Postoperative WHO PS) is not 5
Scan date (at recurrence or last follow-up date if no recurrence)	DD/MM/YYYY	Prompt entry if previous option (Postoperative WHO PS) is not 5
WHO PS at time of recurrence/last follow-up	Dropdown list/checkbox<br><br>• 0<br>• 1<br>• 2<br>• 3<br>• 4	Prompt entry if previous option (Postoperative WHO PS) is not 5
**SRS (SECTION/PAGE 7)**		
Pre-radiation WHO PS	Dropdown list/check box<br><br>• 0<br>• 1<br>• 2<br>• 3<br>• 4	Required entry. Allow one option only
Pre-radiation comorbidity	Check box<br><br>• Hypertension - systolic > 140 or diastolic > 90 and patients on medical treatment<br>• Previous myocardial infarction<br>• Congestive heart failure<br>• Peripheral vascular disease	Required entry. Allow multiple options.<br><br>Note to data collector: Age and comorbidities will be used to calculate the age adjusted Charlson comorbidity index. Each of these is defined in the guide provided

	• Previous stroke/TIA - If hemiplegia present, do not check<br>• Hemi/paraplegia<br>• Diabetes which requires medical treatment<br>• Diabetes with end-organ damage - if so, do not check diabetes that requires treatment<br>• COPD/Asthma<br>• Renal disease<br>• Mild liver disease - Hep B/C or cirrhosis without portal hypertension<br>• Moderate to severe liver disease - cirrhosis with portal hypertension, jaundice, ascites<br>• Peptic ulcer disease<br>• Cancer - excluding basal cell carcinoma<br>• Metastatic cancer - if so, do not check cancer<br>• Rheumatic or connective tissue disease<br>• HIV/AIDS<br>• Skin ulcers/cellulitis<br>• Depression<br>• Dementia<br>• On Warfarin	
Dose	Free field	Required entry
Early CTCAE toxicity (≤3 months)	Checkbox	Required entry

Toxicity	Free field	Prompt entry if previous option (Early CTCAE toxicity (≤3 months)) is ticked<br><br>Note to data collector: https://ctep.cancer.gov/protocoldevelopment/electronic_applications/ctc.htm<br><br>Use the following format: System class organ (SOC)-CTCAE term-grade e.g. nervous system disorders-headache-1. Select the complication with the highest toxicity grade
Late CTCAE toxicity	Checkbox	Required entry
Toxicity	Free field	Prompt entry if previous option (Late CTCAE toxicity) is ticked<br><br>Note to data collector: https://ctep.cancer.gov/protocoldevelopment/electronic_applications/ctc.htm<br><br>Use the following format: System class organ (SOC)-CTCAE term-grade e.g. nervous system disorders-headache-1. Select the complication with the highest toxicity grade
Meningioma progression/regrowth	Checkbox	Required
Scan date (at progression or last follow-up date if no progression)	DD/MM/YYYY	Required
WHO PS at time of progression/last follow-up	Dropdown list/checkbox<br><br>• 0<br>• 1<br>• 2<br>• 3<br>• 4<br>• 5 (dead)	Required. Allow one option only
f**RT (SECTION/PAGE 8)**		

Pre-radiation WHO PS	Dropdown list/check box<br><br>• 0<br>• 1<br>• 2<br>• 3<br>• 4	Required entry
Pre-radiation comorbidity	Check box<br><br>• Myocardial infarction<br>• Congestive heart failure<br>• Peripheral vascular disease<br>• Hemiplegia<br>• Cerebrovascular disease<br>• Pulmonary disease<br>• Diabetes<br>• Renal disease<br>• Liver disease<br>• Peptic ulcer disease<br>• Cancer<br>• Dementia<br>• Connective tissue disease<br>• AIDS<br>• Hypertension<br>• Skin ulcers/cellulitis<br>• Depression<br>• On Warfarin	Required entry. Allow multiple options.<br><br>Note to data collector: Age and comorbidities will be used to calculate the age adjusted Charlson comorbidity index. Each of these is defined in the guide provided
Number of fractions	Free field	Required entry
Fractionated dose	Free field	Required entry
Total dose	Free field	Required entry
Early CTCAE toxicity (≤3 months)	Checkbox	Required entry

Toxicity	Free field	Prompt entry if previous option (Early CTCAE toxicity (≤3 months)) is ticked<br><br>Note to data collector: https://ctep.cancer.gov/protocoldevelopment/electronic_applications/ctc.htm<br><br>Use the following format: System class organ (SOC)-CTCAE term-grade e.g. nervous system disorders-headache-1. Select the complication with the highest toxicity grade
Late CTCAE toxicity	Checkbox	Required entry
Toxicity	Free field	Prompt entry if previous option (Late CTCAE toxicity) is ticked<br><br>Note to data collector: https://ctep.cancer.gov/protocoldevelopment/electronic_applications/ctc.htm<br><br>Use the following format: System class organ (SOC)-CTCAE term-grade e.g. nervous system disorders-headache-1. Select the complication with the highest toxicity grade
Meningioma progression/regrowth	Checkbox	Required
Scan date (at progression or last follow-up date if no progression)	DD/MM/YYYY	Required
WHO PS at time of progression/last follow-up	Dropdown list/checkbox<br><br>• 0<br>• 1<br>• 2<br>• 3<br>• 4<br>• 5 (dead)	Required. Allow one option only
**Discharge from outpatient care/Lost to follow-up (SECTION/PAGE 9)**		

Date of data entry into the database	DD/MM/YYYY	Required entry
Rescanned during the time between discharge/loss to FU and the date of data entry	Checkbox	Required entry
Date of scan	DD/MM/YYYY	Prompt entry if previous option (Readmitted/rescanned during the time between discharge/loss to FU and the date of data entry) has been checked
Reason?	Dropdown list:<br>• Seizure<br>• Headache<br>• Motor<br>• Sensory<br>• Language<br>• Cognitive<br>• Other	Prompt entry if previous option (Readmitted/rescanned during the time between discharge/loss to FU and the date of data entry) has been checked. Allow multiple options
Peritumoural signal intensity on T2	Dropdown list/check box<br>• 0-5%<br>• 6-33%<br>• 34-66%<br>• 67-100%<br>• NA	Prompt entry if previous option (Readmitted/rescanned during the time between discharge/loss to FU and the date of data entry) has been checked. Allow one option only<br>Note to data collector: In relation to tumour volume. If only baseline CT available, NA
Peritumoural signal intensity on FLAIR	Dropdown list:<br>• 0-5%<br>• 6-33%<br>• 34-66%<br>• 67-100%<br>• NA	Prompt entry if previous option (Readmitted/rescanned during the time between discharge/loss to FU and the date of data entry) has been checked. Allow one option only<br>Note to data collector: In relation to tumour volume. If only baseline CT available, NA
Venous sinus nearby	Checkbox<br>• Superior sagittal sinus<br>• Cavernous sinus	Prompt entry if previous option (Readmitted/rescanned during the time between discharge/loss to FU and the date of data entry) has been checked

	• Sigmoid sinus<br>• Transverse sinus<br>• Confluence of sinuses	Note to data collector: Tick box if within 10 mm, in direct contact or invading one of: superior sagittal sinus (SSS), sigmoid sinus (SS), transverse sinus (TS), cavernous sinus (CS) and the confluence of sinuses (CoS)
If yes, specify	Dropdown list/check box	Prompt entry if previous option (venous sinus nearby) has been ticked. Allow one option only
Separate, direct contact or invaded?	Dropdown list/check box<br><br>• Separate<br>• Direct contact<br>• Invaded	Prompt entry if previous option (venous sinus nearby) has been ticked. Allow one option only
Major axis (mm)	Free field	Prompt entry if previous option (Readmitted/rescanned during the time between discharge/loss to FU and the date of data entry) has been checked<br><br>Note to data collector: In mm to 1 dp. Measure on axial T1/CT + GAD
Minor axis (mm)	Free field	Prompt entry if previous option (Readmitted/rescanned during the time between discharge/loss to FU and the date of data entry) has been checked<br><br>Note to data collector: In mm to 1 dp. Perpendicular to the major axis on axial T1/CT + GAD
Cor/sag major axis (mm)	Free field	Prompt entry if previous option (Readmitted/rescanned during the time between discharge/loss to FU and the date of data entry) has been checked<br><br>Note to data collector: In mm to 1 dp. Maximum height
Verdict	Dropdown list/checkbox<br><br>• Related<br>• Unrelated	Prompt entry if previous option (Readmitted/rescanned during the time between discharge/loss to FU and the date of data entry) has been checked. Allow one option only<br><br>Note to data collector: Were the symptoms attributed to the meningioma?
Outcome	Dropdown list/Checkbox	Prompt entry if previous option (Readmitted/rescanned during the time between discharge/loss to FU and the date of data entry) has been checked. Allow one option only

	• Resume follow-up (active monitoring)<br>• Surgery<br>• SRS<br>• *f*RT<br>• Discharge<br>• Lost to follow-up<br>• Dead	Note to data collector: You will be directed to the relevant page/section dependent on the choice. If the patient died or was lost to follow-up after this interval scan, please choose one of the last two options, albeit the intention might have been to continue monitoring or intervene. If the option you choose is set to take place in the future, choose the option, save the data entered and exit the form with no further information required.
Overall outcome	Dropdown list/Checkbox:<br><br>• Dead<br>• Alive	Prompt entry if previous option (Rescanned during the time between discharge/loss to FU and the date of data entry) is not ticked.<br><br>Allow one option only<br><br>Note to data collector: You will be directed to section 10 if Dead is selected.
**Mortality (SECTION/PAGE 10)**		
Date of death	DD/MM/YYYY	Required entry
Cause of death	Dropdown list/checkbox<br><br>• Meningioma-related<br>• Unrelated	Required entry. Allow one option only<br><br>Note to data collector: An example of a meningioma-related death would be for example status epilepticus in a patient who manifested seizures but didn't have treatment. An unrelated death would be for example a community acquired pneumonia. For the purpose of the study, any death occurring from a morbidity, which did not necessitate neurosurgical input/opinion will be classified as unrelated

1.2. Appendix. 2. ICOM classification of meningioma location

ICOM classification system of meningioma locations			
**Main category**	Subcategories		
**Convexity**	Anterior [1]	Posterior[1]	
**Parasagittal**	Anterior[1]	Posterior[1]	Falco-tentorial
**Parafalcine**	Anterior[1]	Posterior[1]	Falco-tentorial
**Sphenoid wing**	Lateral	Medial (including ACP)	
**Anterior midline**	Cribriform plate or olfactory groove[2]	Planum[3]	Tuberculum and diaphragma sellae
**Posterior fossa - midline**	Clival	Petro-clival	Anterior foramen magnum[4]
**Posterior fossa – Lateral & posterior**	Petrous	Squamous occipital	Posterior foramen magnum[4]
**Tentorial**	Supratentorial	Infratentorial	
**Intraventricular**			
**Pineal region[5]**			

[1] The main attachment is located anterior or posterior, respectively, to the coronal suture
[2] Arising between the crista galli and the fronto-sphenoid suture
[3] Arising between the fronto-sphenoid suture and the limbus sphenoidale
[4] The main attachment is located anterior or posterior, respectively, to the hypoglossal canal
[5] No obvious tentorial attachment

For peer review only

TRIPOD Checklist: Prediction Model Validation

Section/Topic	Item	Checklist Item	Page
**Title and abstract**			
Title	1	Identify the study as developing and/or validating a multivariable prediction model, the target population, and the outcome to be predicted.	1
Abstract	2	Provide a summary of objectives, study design, setting, participants, sample size, predictors, outcome, statistical analysis, results, and conclusions.	2
**Introduction**			
Background and objectives	3a	Explain the medical context (including whether diagnostic or prognostic) and rationale for developing or validating the multivariable prediction model, including references to existing models.	4,5
	3b	Specify the objectives, including whether the study describes the development or validation of the model or both.	5
**Methods**			
Source of data	4a	Describe the study design or source of data (e.g., randomized trial, cohort, or registry data), separately for the development and validation data sets, if applicable.	6
	4b	Specify the key study dates, including start of accrual; end of accrual; and, if applicable, end of follow-up.	6
Participants	5a	Specify key elements of the study setting (e.g., primary care, secondary care, general population) including number and location of centres.	6,7
	5b	Describe eligibility criteria for participants.	6,7
	5c	Give details of treatments received, if relevant.	10,11
Outcome	6a	Clearly define the outcome that is predicted by the prediction model, including how and when assessed.	7
	6b	Report any actions to blind assessment of the outcome to be predicted.	N/A
Predictors	7a	Clearly define all predictors used in developing or validating the multivariable prediction model, including how and when they were measured.	9
	7b	Report any actions to blind assessment of predictors for the outcome and other predictors.	N/A
Sample size	8	Explain how the study size was arrived at.	7
Missing data	9	Describe how missing data were handled (e.g., complete-case analysis, single imputation, multiple imputation) with details of any imputation method.	N/A
Statistical analysis methods	10c	For validation, describe how the predictions were calculated.	12
	10d	Specify all measures used to assess model performance and, if relevant, to compare multiple models.	12,13
	10e	Describe any model updating (e.g., recalibration) arising from the validation, if done.	13
Risk groups	11	Provide details on how risk groups were created, if done.	N/A
Development vs. validation	12	For validation, identify any differences from the development data in setting, eligibility criteria, outcome, and predictors.	N/A
**Results**			
Participants	13a	Describe the flow of participants through the study, including the number of participants with and without the outcome and, if applicable, a summary of the follow-up time. A diagram may be helpful.	N/A
	13b	Describe the characteristics of the participants (basic demographics, clinical features, available predictors), including the number of participants with missing data for predictors and outcome.	N/A
	13c	For validation, show a comparison with the development data of the distribution of important variables (demographics, predictors and outcome).	N/A
Model performance	16	Report performance measures (with CIs) for the prediction model.	N/A
Model-updating	17	If done, report the results from any model updating (i.e., model specification, model performance).	N/A
**Discussion**			
Limitations	18	Discuss any limitations of the study (such as nonrepresentative sample, few events per predictor, missing data).	N/A
Interpretation	19a	For validation, discuss the results with reference to performance in the development data, and any other validation data.	N/A
	19b	Give an overall interpretation of the results, considering objectives, limitations, results from similar studies, and other relevant evidence.	N/A
Implications	20	Discuss the potential clinical use of the model and implications for future research.	N/A
**Other information**			
Supplementary information	21	Provide information about the availability of supplementary resources, such as study protocol, Web calculator, and data sets.	N/A
Funding	22	Give the source of funding and the role of the funders for the present study.	21

We recommend using the TRIPOD Checklist in conjunction with the TRIPOD Explanation and Elaboration document.

For peer review only - http://bmjopen.bmj.com/site/about/guidelines.xhtml
